# Subgroups of patients with young-onset type 2 diabetes in India reveal insulin deficiency as a major driver

**DOI:** 10.1007/s00125-021-05543-y

**Published:** 2021-10-23

**Authors:** Rashmi B. Prasad, Olof Asplund, Sharvari R. Shukla, Rucha Wagh, Pooja Kunte, Dattatrey Bhat, Malay Parekh, Meet Shah, Sanat Phatak, Annemari Käräjämäki, Anupam Datta, Sanjeeb Kakati, Tiinamaija Tuomi, Banshi Saboo, Emma Ahlqvist, Leif Groop, Chittaranjan S. Yajnik

**Affiliations:** 1 Department of Clinical Sciences, Diabetes and Endocrinology, CRC, Lund University, Malmö, Sweden; 2 Diabetes Unit, Kamalnayan Bajaj Diabetology Research Centre, King Edward Memorial Hospital and Research Centre, Pune, India; 3 Symbiosis Statistical Institute, Symbiosis International University, Pune, India; 4 Dia Care - Diabetes Hormone Clinic, Ahmedabad, India; 5 Department of Primary Health Care, Vaasa Central Hospital, Vaasa, Finland; 6 Diabetes Center, Vaasa Health Care Center, Vaasa, Finland; 7 Assam Medical College and Hospital, Dibrugarh, India; 8 Abdominal Center, Endocrinology, Helsinki University Central Hospital, Research Program for Clinical and Molecular Metabolism, University of Helsinki, Helsinki, Finland; 9 Folkhälsan Research Center, Helsinki, Finland; 10 Institute for Molecular Medicine Finland FIMM, Helsinki University, 00290 Helsinki, Finland

**Keywords:** Europe, India, Insulin deficiency, Subgroups, Type 2 diabetes, Young-onset type 2 diabetes

## Abstract

**Aim/hypothesis:**

Five subgroups were described in European diabetes patients using a data driven machine learning approach on commonly measured variables. We aimed to test the applicability of this phenotyping in Indian individuals with young-onset type 2 diabetes.

**Methods:**

We applied the European-derived centroids to Indian individuals with type 2 diabetes diagnosed before 45 years of age from the WellGen cohort (*n* = 1612). We also applied de novo *k*-means clustering to the WellGen cohort to validate the subgroups. We then compared clinical and metabolic-endocrine characteristics and the complication rates between the subgroups. We also compared characteristics of the WellGen subgroups with those of two young European cohorts, ANDIS (*n* = 962) and DIREVA (*n* = 420). Subgroups were also assessed in two other Indian cohorts, Ahmedabad (*n* = 187) and PHENOEINDY-2 (*n* = 205).

**Results:**

Both Indian and European young-onset type 2 diabetes patients were predominantly classified into severe insulin-deficient (SIDD) and mild obesity-related (MOD) subgroups, while the severe insulin-resistant (SIRD) and mild age-related (MARD) subgroups were rare. In WellGen, SIDD (53%) was more common than MOD (38%), contrary to findings in Europeans (Swedish 26% vs 68%, Finnish 24% vs 71%, respectively). A higher proportion of SIDD compared with MOD was also seen in Ahmedabad (57% vs 33%) and in PHENOEINDY-2 (67% vs 23%). Both in Indians and Europeans, the SIDD subgroup was characterised by insulin deficiency and hyperglycaemia, MOD by obesity, SIRD by severe insulin resistance and MARD by mild metabolic-endocrine disturbances. In WellGen, nephropathy and retinopathy were more prevalent in SIDD compared with MOD while the latter had higher prevalence of neuropathy.

**Conclusions /interpretation:**

Our data identified insulin deficiency as the major driver of type 2 diabetes in young Indians, unlike in young European individuals in whom obesity and insulin resistance predominate. Our results provide useful clues to pathophysiological mechanisms and susceptibility to complications in type 2 diabetes in the young Indian population and suggest a need to review management strategies.

**Graphical abstract:**

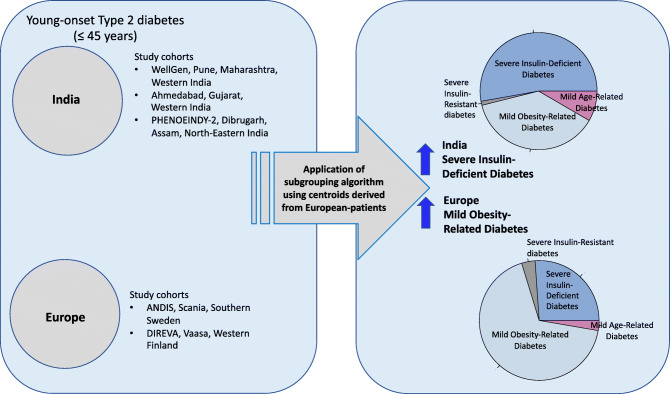

**Supplementary Information:**

The online version contains peer-reviewed but unedited supplementary material available at 10.1007/s00125-021-05543-y.

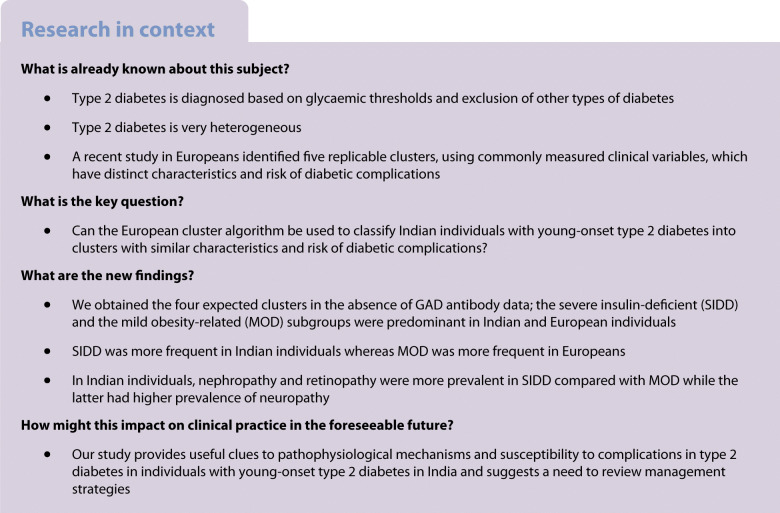



## Introduction

Type 2 diabetes has been traditionally considered as one disease characterised by both insulin resistance and insulin deficiency. Nevertheless, the disease is heterogeneous [1]. A formal description of five distinct subgroups was proposed in a large Swedish cohort [2] and replicated in other populations [2–5]: severe autoimmune diabetes (SAID) severe insulin-deficient diabetes (SIDD); severe insulin-resistant diabetes (SIRD); mild obesity-related diabetes (MOD); and mild age-related diabetes (MARD). The subgroups differed not only in their clinical characteristics at diagnosis but also in their pathophysiological mechanisms and susceptibility to complications.

India is referred to as one of the diabetes capitals of the world, and Indian individuals with type 2 diabetes differ from Europeans in that they develop diabetes at a younger age and are thinner [6, 7]. Indians also differ in body composition, having higher fat and lower lean proportions at the same BMI [8]. Given the role of adiposity in insulin resistance, it has therefore been assumed that type 2 diabetes in Indians is primarily driven by insulin resistance [9]. However, it is increasingly recognised that insulin deficiency may be a significant driver of diabetes in Indians [10]. Recent studies show that both the increase in the diabetes prevalence and the characteristics of the affected individuals vary in different parts of India [11, 12]. Lean type 2 diabetes is prevalent in India, especially in undernourished regions [13]. Recent studies have shown that subgroups of type 2 diabetes in Indians show partial concordance with those in Europeans [14, 15].

Type 2 diabetes is diagnosed at a younger age in India and its pathophysiology and heterogeneity warrants further investigation. Younger age at diagnosis has distinct implications for treatment, long-term complications and mortality as well as socioeconomic burden [16]. Therefore, early identification of subclasses may be vital for appropriate treatment to reduce adverse outcomes [17]. To address this, we implemented the Swedish algorithm [2] to identify subgroups of young Indians with type 2 diabetes diagnosed before 45 years of age from the WellGen cohort from Pune, India [18]. We then compared Indian and European type 2 diabetes subgroups to obtain information on the relative distributions and characteristics in the two populations. We also performed de novo clustering of individuals from the WellGen study to assess whether clusters obtained were similar in proportion and characteristics to those from the European-derived centroids. Finally, we investigated the subclassification of type 2 diabetes in cohorts from two other geographical regions across India.

## Methods

### Study population

#### WellGen (Pune, Maharashtra, western India)

The WellGen study includes patients visiting the Diabetes Unit, KEM Hospital, Pune and associated clinics for routine diabetes management between 2004 and 2006 [18]. Individuals diagnosed with diabetes below 45 years of age using the WHO guidelines were included [1]. Diagnosis of type 2 diabetes was based on clinical criteria: age at diagnosis >20 years; no history of ketoacidosis; central obesity (waist–hip ratio: WHR >0.80 in women and >0.90 in men); and response to treatment with oral glucose-lowering agents. Individuals with a clinical diagnosis of type 1 diabetes (diagnosis before 20 years of age, history of ketoacidosis, continuous insulin treatment since diagnosis), fibrocalculous pancreatic diabetes (FCPD) or fulfilling criteria for monogenic diabetes were excluded. In total, 1612 individuals were included (Table 1).

**Table 1:** Clinical and biochemical characteristics of participants enrolled in WellGen and ANDIS study with age at diagnosis less than 45 years

Characteristic	WellGen			ANDIS			WellGen vs ANDIS		
	Male participants	Female participants	All	Male participants	Female participants	All	*p* value (male participants)	*p* value (female participants)	*p* value (all)
No. of participants	902	710	1612	567	395	962			
Age at diabetes diagnosis, years	37.39 (5.58)	36.47 (5.99)	36.98 (5.78)	39.13 (5.8)	38.4 (6.16)	38.83 (5.96)	<0.0001^a^	<0.0001^a^	<0.0001^a^
BMI, kg/m^2^	25.43 (3.74)	27.14 (4.27)	26.18 (4.07)	32.47 (6.60)	33.66 (7.30)	32.96 (6.92)	<0.0001^a^	<0.0001^a^	<0.0001^a^
Fasting glucose, mmol/l	9.08 (3.23)	9.48 (3.45)	9.26 (3.33)	9.28 (3.36)	8.34 (2.80)	8.89 (3.18)	0.23	<0.0001^a^	0.007^a^
HbA_1c_, mmol/mol	73.23 (23.04)	71.62 (22.19)	72.52 (22.68)	75.09 (25.55)	60.93 (24.03)	69.28 (25.88)	0.16	<0.0001^a^	0.0008^a^
HbA_1c_, %	8.8 (4.2)	8.7 (4.2)	9.1 (4.2)	9.0 (4.5)	7.7 (4.4)	8.5 (4.5)	0.16	<0.0001^a^	0.0008^a^
Fasting C-peptide, nmol/l	0.76 (0.46)	0.76 (0.45)	0.76 (0.46)	1.20 (0.57)	1.97 (0.56)	1.20 (0.56)	<0.0001^a^	<0.0001^a^	<0.0001^a^
HOMA2-B	58.7 (40.28)	56.39 (41.93)	57.69 (41.02)	81.09 (50.59)	88.36 (45.48)	84.11 (48.91)	<0.0001^a^	<0.0001^a^	<0.0001^a^
HOMA2-IR	2.12 (1.38)	2.15 (1.35)	2.13 (1.37)	3.27 (1.62)	3.12 (1.51)	3.21 (1.58)	<0.0001^a^	<0.0001^a^	<0.0001^a^

Values are mean (SD)

^a^Bonferroni corrected significant *p* values

*p* values were calculated using *t* test

Clinical information including age, sex, age at diabetes diagnosis, family history and socioeconomic status was obtained through a standardised questionnaire. Height, weight, waist and hip circumferences, and BP were measured using standardised methods [18, 19]. Fasting plasma glucose, total cholesterol, HDL-cholesterol, triacylglycerols and HbA_1c_ were measured using standard laboratory assays as described previously [18, 19]. Fasting C-peptide was measured by ELISA (Diagnostic Biochem Canade, ON, Canada). Fasting glucose and C-peptide measurements were used to calculate Homeostatic model assessment 2 estimates of β-cell function (HOMA2-B) and insulin resistance (HOMA2-IR) values [20, 21]. Details of treatment (insulin, oral glucose-, BP- and lipid-lowering medication) were recorded.

Cardiovascular complications were defined by ICD-10 codes (http://apps.who.int/classifications/icd10/browse/2016/en): Coronary artery disease (CAD) I20–21, I24, I251 and I253–259; stroke I60–61 and I63–64. Nephropathy was diagnosed by urine strip albumin measurement (nil, trace, and +), and by eGFR calculation (ml min^−1^ [1.73 m body surface area]^−2^) by the Modification of Diet in Renal Disease (MDRD) formula (>90 ml min^−1^ [1.73 m]^−2^ normal; and 90–60 ml min^−1^ [1.73 m]^−2^ mild, 60–30 ml min^−1^ [1.73 m]^−2^ moderate and <30 ml min^−1^ [1.73 m]^−2^ severe chronic kidney disease [CKD]). Diagnosis of retinopathy was based on dilated fundus examination performed by an ophthalmologist and was classified as non-proliferative diabetic retinopathy or proliferative diabetic retinopathy. Peripheral neuropathy was diagnosed by biothesiometer (non-perception of vibration sense at 15 or higher amperes at two or more sites on the feet) (Table 2).

**Table 2:** Complications and current treatment for participants of the WellGen and ANDIS studies stratified by sex

Complication/treatment	WellGen				ANDIS			
	Male participants	Female participants	Total (%)	*p* value	Male participants	Female participants	Total (%)	*p* value
*n* (%)	902 (56.0)	710 (44.0)	1612 (100)		567 (58.9)	395 (41.1)	962 (100)	
Duration of diabetes, years	9.73 (8.57)	9.70 (7.74)	9.72 (8.21)		4.27 (2.56)	4.27 (2.34)	4.27 (2.47)	
Current treatment								
Diet only	77 (8.5)	58 (8.2)	135 (8.4)	0.791	34 (6.0)	43 (10.9)	77 (8.0)	0.006^a^
OHA only	558 (61.9)	421 (59.3)	979 (60.7)	0.295	382 (67.4)	247 (62.5)	629 (65.4)	0.121
Only on insulin	51 (5.7)	34 (4.8)	85 (5.3)	0.440	28 (4.9)	31 (7.8)	59 (6.1)	0.064
Both OHA and insulin	216 (23.9)	197 (27.7)	413 (25.6)	0.083	122 (21.5)	72 (18.2)	194 (20.2)	0.211
Complications								
CVD (Coronary events and/or stroke)^b^	89 (9.9)	31 (4.4)	120 (7.4)	0.0001^a^	27 (4.8)	7 (1.8)	34 (3.6)	0.013
Coronary event	76 (8.4)	24 (3.3)	100 (6.2)	0.0001^a^	19 (3.4)	4 (1.0)	23 (2.4)	0.019
Stroke	17 (1.9)	9 (1.3)	26 (1.7)	0.329	8 (1.4)	3 (0.8)	11 (1.2)	0.348
Nephropathy (proteinuria and/or CKD)^c^	361 (40.0)	200 (28.2)	561 (34.8)	0.0001^a^	181 (45.9)	129 (45.7)	310 (45.9)	0.960
Macroalbuminuria^d^	167 (19.2)	83 (12.3)	250 (16.2)	0.0001^a^	61 (17.1)	29 (11.4)	90 (14.7)	0.047
CKD: eGFR^e, f^	268 (32.3)	145 (22.7)	413 (28.1)	0.0001^a^	142 (26.0)	109 (28.7)	251 (27.1)	0.359
Early CKD (60–90 ml min^−1^ [1.73m]^−2^)	208 (25.0)	122 (19.1)	330 (22.4)	0.007	132 (24.6)	105 (27.9)	237 (26.0)	0.257
Moderate (30–60 ml min^−1^ [1.73m]^−2^)	54 (6.5)	20 (3.1)	74 (5.0)	0.003^a^	7 (1.3)	4 (1.1)	11 (1.2)	0.747
Severe (<30 ml min^−1^ [1.73m]^−2^)	6 (0.7)	3 (0.5)	9 (0.6)	0.537	3 (0.5)	0 (0.0)	3 (0.3)	0.148
Retinopathy (*n*=657)^g^	102 (29.4)	78 (25.2)	180 (27.4)	0.225	34 (19.4)	23 (20.0)	57 (19.8)	0.905
NPDR	93 (26.8)	74 (23.9)	167 (25.4)	0.389	33 (19.0)	22 (19.3)	55 (19.1)	0.944
PDR	9 (2.6)	4 (1.3)	13 (2.0)	0.231	1 (0.6)	1 (0.9)	2 (0.7)	0.764
Neuropathy^h^	359 (40.7)	351 (50.6)	710 (45.1)	0.0001^a^	14 (2.5)	2 (0.5)	16 (1.7)	0.019

Values are *n* (%)

^a^Bonferroni corrected significant *p* values

^b^CVD: data available for *n*=1612 (Male *n*=902, Female *n*=710) for WellGen, *n*=956 (Male *n*=563, Female *n*=393) for ANDIS

^c^Nephropathy: data available for *n*=1612 (Male *n*=902, Female *n*=710) for WellGen, *n*=676 (Male *n*=394, Female *n*=282) for ANDIS

^d^Macroalbuminuria: data available for *n*=1544 (Male *n*=869, Female *n*=675) for WellGen, *n*=611(Male *n*=356, Female *n*=255) for ANDIS

^e^Based on MDRD formula

^f^CKD: eGFR: data available for *n*=1471(Male *n*=831, Female *n*=640) for WellGen and *n*=927 (Male *n*=547, Female *n*=380) for ANDIS

^g^Diabetic retinopathy: data available for *n*=657 (Male *n*=347, Female *n*=310) for WellGen and *n*=288 (Male *n*=174, Female *n*=114) for ANDIS

^h^Neuropathy: data available for *n*=1576 (Male *n*=883, Female *n*=693), diagnosed using Biothesiometry for WellGen, *n*=956 (Male *n*=563, Female *n*=393), ICD codes ICD-10 = E104 or E114 for ANDIS

*p* values were calculated by χ^2^ test

NPDR, non-proliferative diabetic retinopathy; OHA, oral glucose-lowering agent; PDR, proliferative diabetic retinopathy

#### Ahmedabad (Gujarat, western India)

Patients with a clinical diagnosis of type 2 diabetes visiting the DiaCare Clinic, Ahmedabad during 2018–2019 diagnosed below 45 years of age and duration of diabetes less than 2 years were invited to participate and 187 individuals consented. Measurements included anthropometry, HbA_1c_ levels and a fasting measurement of plasma glucose and C-peptide (MAGLUMI C-peptide; CLIA, Shenzhen, China) (electronic supplementary material [ESM] Table 1).

#### PHENOEINDY-2 (Dibrugarh, Assam, North-east India)

Patients with clinical diagnosis of type 2 diabetes attending the medical outpatient department of Assam Medical College, Dibrugarh during 2017–2019 if diagnosed below 40 years of age were invited to participate and 205 individuals consented. Measurements included anthropometry, HbA_1c_ levels, a fasting measurement of plasma glucose and C-peptide (ELISA, Diagnostic Biochem Canade, ON, Canada) (ESM Table 2).

#### ANDIS (Scania, Southern Sweden)

The ANDIS project comprises newly diagnosed diabetic individuals aged >18 years in Scania County, Sweden between 2008 and 2016 [2]. Biochemical and anthropometric measurements and presence of complications were recorded as described elsewhere [2]. For the current study, 962 individuals diagnosed with type 2 diabetes before 45 years of age were included. We excluded any person with known type 1 diabetes, monogenic diabetes and GAD antibody positivity (so-called SAID) to maintain concordance with the WellGen study. The prevalence of complications (diagnosed as described previously [2]; Table 2) was recorded ~4.2 years after diagnosis.

#### DIREVA (Vaasa, Western Finland)

DIREVA includes 5107 individuals with diabetes recruited from 2009 to 2014 in the Vaasa Hospital District. For the current study, 424 individuals with type 2 diabetes diagnosed below 45 years of age were included; exclusion criteria were similar to those for ANDIS (ESM Table 3). Biochemical and anthropometric measurements have been described elsewhere [2]. No treatment or complication data from DIREVA have been included in the current study.

### Ethics statement

All studies were approved by the local/regional Institutional Ethics Committees, and all participants gave written informed consent.

### Statistical methods

Participants with measurements above or below 5 SDs from the mean for the clustering parameters were excluded from the analysis and values outside the limits for HOMA2 calculation (fasting glucose <3 mmol/l or >25 mmol/l; C-peptide <0.2 ng/ml or >3.5 ng/ml) were capped to the proximal upper or lower limits. To perform supervised clustering in relation to the European-derived cluster coordinates, phenotypes (age at diagnosis, HbA_1c_, HOMA2-B, HOMA2-IR and BMI) were scaled using the same scaling parameters (mean and SD) as described previously [2]. Due to the unavailability of GAD autoantibody data in the Indian study (WellGen), we only included clusters 2–5 (SIDD, SIRD, MOD and MARD). Participants were assigned to the predetermined clusters on the basis of which ANDIS cluster they were most similar to, calculated as their Euclidean distance from the nearest cluster centre derived from ANDIS coordinates. The nearest centroid method was used to find the nearest centroid (as measured with Euclidean distances) for each individual. This resulted in each participant being assigned to any of the four clusters: 2/SIDD; 3/SIRD; 4/MOD; or 5/MARD. Given the wide range of duration of diabetes in WellGen, we performed a sensitivity analysis by separately assessing the type 2 diabetes subgroups among those within 5 years of diagnosis and those above.

To perform unsupervised clustering, all previously mentioned variables were used in a separate analysis. Given that the results from the supervised clustering analysis showed a strong bias towards the 2/SIDD and 4/MOD cluster, and the silhouette analysis indicated that two was the most stable number of clusters, we performed *k*-means clustering into two clusters. All phenotypes were scaled to have a mean of 0 and an SD of 1, this time with scaling parameters derived from the data itself. *k*-means clustering was then performed separately for female and male participants using the *k*-means runs algorithm from the fpc package version 2.1–11.1 (https://CRAN.R-project.org/package=fpc) in R version 3.4 [22].

We investigated differences between groups (male and female participants or between four subgroups) by *t* test or ANOVA for clinical characteristics and by χ^2^ test for complications. To compare differences in the rate of complications between subgroups, we also used logistic regression with adjustment for age at diagnosis, sex and duration of diabetes. We used SPSS version 22.0 for these analyses.

### Type 1 diabetes genetic risk scores

In the absence of GAD autoantibody data, we applied a previously validated ‘type 1’ genetic risk score (GRS) (ESM Table 4) to 560 WellGen participants with available data to estimate the proportion of those carrying autoimmune risk alleles. The characteristics of these individuals did not differ from the characteristics of those for whom genotyping was not available (ESM Table 5). A positive control group comprised 261 individuals with type 1 diabetes, as described previously [23]. A negative control group comprised 461 participants with normal glucose tolerance (75 g OGTT; WHO 1999 criteria) from the Pune Maternal Nutrition Study (PMNS) [24].

### Genotyping

Genome-wide genotyping data was generated on WellGen and PMNS participants using Affymetrix SNP 6.0 Chips (Affymetrix, CA, USA) and the Infinium Global Screening Array V1 B37 (Illumina, San Diego, CA, USA) for the type 1 diabetes cohort. Quality control and imputation were performed as described in ESM Methods.

### GRS

A previously described set of nine SNPs was used for type 1 diabetes GRS calculations (ESM Table 4) [23, 25, 26]. In the absence of genotyping data for rs7454108, a proxy SNP rs3957146 (linkage disequilibrium [LD]: *r*^2^ = 1, D′ = 1) was used. LD is the non-random association of alleles at multiple DNA markers resulting from their close proximity to one another within a chromosome and are therefore inherited together. Classical LD measures include D′ and *r*^2^, where D′ and *r*^2^ values >0.8 indicate a higher degree of co-inheritance. The haplotype was constructed using rs2187668 + rs3957146 as described previously [23] and GRS scores were computed on PLINK 1.09 (http://pngu.mgh.harvard.edu/~purcell/plink/) [27] using weighted scores. Logistic regression was performed to assess the discriminatory power of GRS between type 1 diabetes and other subgroups.

## Results

We first sought to investigate the subgroups of individuals with young-onset type 2 diabetes in the Indian WellGen study and compare them with Swedish subgroups from the ANDIS study.

The WellGen study comprised individuals diagnosed with type 2 diabetes before 45 years of age, and all relevant data required for clustering were available for 1624 participants. After applying exclusion criteria, 1612 individuals (56% men) with mean age at diagnosis of 37 years, duration of diabetes ~10 years and BMI 26.18 kg/m^2^ were included (Table 1 and ESM Fig. 1).

For comparison, we selected 962 participants with type 2 diabetes (58.9% men, mean age at diagnosis 38.83 years) from the ANDIS study, after excluding 577 individuals belonging to cluster 1 (SAID) [2]. The Indian participants were younger at diagnosis, had lower BMI, higher fasting plasma glucose and lower fasting C-peptide, HOMA2-B and HOMA2-IR compared with the ANDIS sub-cohort (Table 1). The proportion of participants receiving lifestyle management alone, glucose-lowering oral agents and insulin treatment was broadly similar in both cohorts (Table 2). In the WellGen study, men had a higher prevalence of cardiovascular events and nephropathy compared with women, whereas the prevalence of neuropathy was higher in women. There was no difference with respect to these complications between men and women in the ANDIS study (Table 2). We did not compare the complication rates between the two cohorts because of the difference in duration of diabetes.

### SIDD predominates in India, MOD in Sweden

**Fig. 1:**
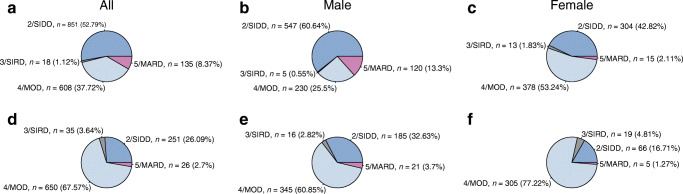
Distribution of participants from the WellGen and ANDIS study in the predefined clusters. (**a–c**) Distribution of WellGen participants, showing all participants (*n*=1612) (**a**), men with diabetes (*n*=902) (**b**) and women with diabetes (*n*=710) (**c**). (**d–f**) Distribution of ANDIS participants, showing all participants (*n*=962) (**d**), men with diabetes (*n*=567) (**e**) and women with diabetes (*n*=395) (**f**)

In the absence of GAD autoantibody data, we obtained the four expected clusters, albeit with different proportions. In the WellGen study, the SIDD cluster was the largest subgroup (52.8%), followed by the mild obesity-related MOD (37.7%), while severe insulin-resistant SIRD (1.1%) and mild age-related MARD (8.4%) were less common (Fig. 1, Table 3). In a sensitivity analysis, with increasing duration of diabetes (from <5 years to >5 years), the proportion of participants in the SIDD subgroup increased (from 45.5% to 56.9%) while that in MOD group decreased (from 44.7% to 33.8%) (ESM Table 6).

**Table 3:** Characteristics of participants enrolled in WellGen and ANDIS study by clusters for all participants, and for male and female participants

Characteristic	WellGen						ANDIS						WellGen vs ANDIS	
	SIDD	SIRD	MOD	MARD	*p* value	*p*1 value	SIDD	SIRD	MOD	MARD	*p* value	*p*1 value	*p* value for SIDD	*p* value for MOD
All participants (*N* = 1612 for WellGen, *N* = 962 for ANDIS)														
Participants in subroup, *n* (%)	851 (52.79)	18 (1.12)	608 (37.72)	135 (8.37)			251 (26.09)	35 (3.64)	650 (67.57)	26 (2.7)				
Age at diagnosis, years	36.86 (5.81)	38.83 (4.93)	36.25 (5.77)	40.84 (3.98)	0.005^*a*^	<0.0001^a^	38.98 (6.24)	39.94 (5.87)	38.56 (5.9)	42.5 (2.79)	<0.0001^a^	<0.0001^a^	<0.0001^a^	<0.0001^a^
Duration of diabetes, years	10.49 (8.25)	6.41 (7.16)	8.72 (8.03)	9.83 (8.44)	0.001^a^	--	--	--	--	--	--	--	--	--
BMI, kg/m^2^	25.02 (3.45)	28.37 (5.27)	28.4 (4.03)	23.22 (2.44)	<0.0001^a^	<0.0001^a^	28.29 (5.43)	36.62 (6.28)	34.92 (6.41)	24.11 (2.33)	<0.0001^a^	<0.0001^a^	<0.0001^a^	<0.0001^a^
Fasting glucose, mmol/l	10.77 (3.39)	6.86 (1.89)	7.85 (2.37)	6.50 (1.47)	<0.0001^a^	<0.0001^a^	11.75 (3.67)	7.05 (2.22)	7.97 (2.25)	6.75 (1.26)	<0.0001^a^	<0.0001^a^	<0.0001^a^	0.06
HbA_1c_, mmol/mol	87.01 (19.34)	56.7 (13.1)	58.33 (12.92)	47.25 (10.37)	<0.0001^a^	<0.0001^a^	100.6 (19.62)	55.19 (13.79)	58.87 (17.64)	45.85 (7.41)	<0.0001^a^	<0.0001^a^	<0.0001^a^	0.53
HbA_1c_, %	10.1 (3.9)	7.3 (3.3)	7.5 (3.3)	6.5 (3.1)	<0.0001^a^	<0.0001^a^	11.4 (4.0)	7.2 (3.4)	7.5 (3.8)	6.3 (2.8)	<0.0001^a^	<0.0001^a^	<0.0001^a^	0.53
Fasting C-peptide, nmol/l	0.68 (0.39)	1.01 (0.62)	0.89 (0.48)	0.58 (0.29)	<0.0001^a^	<0.0001^a^	0.85 (0.4)	2.53 (0.61)	1.29 (0.48)	0.73 (0.24)	<0.0001^a^	<0.0001^a^	<0.0001^a^	<0.0001^a^
HOMA2-B	37.66 (22.24)	214.99 (41.01)	77.49 (42.02)	73.74 (31.16)	<0.0001^a^	<0.0001^a^	41.03 (28.34)	968.71 (56.43)	94.79 (38.53)	80.15 (27.62)	<0.0001^a^	<0.0001^a^	0.048	<0.0001^a^
HOMA2-IR	2.08 (1.41)	4.73 (1.56)	2.28 (1.29)	1.42 (0.76)	0.314	<0.0001^a^	2.67 (1.42)	6.17 (1.86)	3.31 (1.42)	1.78 (0.63)	<0.0001^a^	<0.0001^a^	0.043	<0.0001^a^
Male participants (*n* = 902 for WellGen, *n* = 567 for ANDIS)														
Participants in subgroup, *n* (%)	547 (60.6)	5 (0.55)	230 (25.50)	120 (13.30)			185 (32.6)	16 (2.82)	345 (60.85)	21 (3.7)				
Age at diagnosis, years	36.96 (5.67)	42.07 (10.27)	36.59 (5.43)	40.7 (4.12)	<0.0001^a^	<0.0001^a^	38.91 (6.08)	40.56 (6.19)	39.0 (5.73)	42.05 (2.87)	0.08	0.089	<0.0001^a^	<0.0001^a^
Duration of diabetes, years	10.55 (8.70)	8.03 (6.59)	7.71 (7.98)	9.98 (8.56)	0.005^a^	--								
BMI, kg/m^2^	24.44 (3.08)	28.75 (3.54)	28.77 (3.67)	23.41 (2.34)	<0.0001^a^	<0.0001^a^	27.45 (3.9)	36.74 (6.01)	35.44 (5.9)	24.72 (2.11)	<0.0001^a^	<0.0001^a^	<0.0001^a^	<0.0001^a^
Fasting glucose, mmol/l	10.29 (3.22)	5.75 (1.0)	7.63 (2.43)	6.52 (1.53)	<0.0001^a^	<0.0001^a^	11.80 (3.5)	7.58 (3.04)	8.16 (2.53)	6.77 (2.53)	<0.0001^a^	<0.0001^a^	<0.0001^a^	0.013
HbA_1c_, mmol/mol	84.67 (20.57)	49.07 (10.27)	59.94 (13.35)	47.55 (10.02)	<0.0001^a^	<0.0001^a^	99.58 (20.31)	57.33 (13.82)	64.59 (18.46)	45.30 (7.11)	<0.0001^a^	<0.0001^a^	<0.0001^a^	0.001^a^
HbA_1c_, %	9.9 (4.0)	6.6 (3.1)	7.6 (3.3)	6.5 (3.1)	<0.0001^a^	<0.0001^a^	11.3 (4.0)	7.4 (3.4)	8.1 (3.8)	6.3 (2.8)	<0.0001^a^	<0.0001^a^	<0.0001^a^	0.001^a^
Fasting C-peptide, nmol/l	0.66 (0.38)	2.17 (0.44)	1.08 (0.51)	0.60 (0.30)	<0.0001^a^	<0.0001^a^	0.82 (0.35)	2.65 (0.61)	1.36 (0.49)	0.72 (0.49)	<0.0001^a^	<0.0001^a^	<0.0001^a^	<0.0001^a^
HOMA2-B	39.33 (22.5)	229.16 (40.98)	92.27 (42.49)	75.58 (31.92)	<0.0001^a^	<0.0001^a^	39.15 (25.31)	200.56 (74.37)	98.19 (40.98)	78.57 (27.41)	<0.0001^a^	<0.0001^a^	0.92	0.094
HOMA2-IR	1.96 (1.34)	4.99 (1.23)	2.76 (1.41)	1.47 (0.78)	0.092	<0.0001^a^	2.59 (1.27)	6.67 (2.23)	3.56 (1.48)	1.77 (0.65)	<0.0001^a^	<0.0001^a^	<0.0001^a^	<0.0001^a^
Female participants (*n* = 710 for WellGen, *n* = 395 for ANDIS)														
Participants in subgroup, *n* (%)	304 (42.82)	13 (1.83)	378 (53.24)	15 (2.11)			66 (16.7)	19 (4.8)	305 (77.2)	5 (1.26)				
Age at diagnosis, years	36.68 (6.06)	37.51 (4.92)	36.05 (5.96)	41.98 (2.51)	0.722	0.001^a^	39.2 (6.72)	39.42 (5.71)	38.07 (6.06)	44.4 (1.34)	0.03	0.045	0.0028^a^	<0.0001^a^
Duration of diabetes, years	10.38 (7.37)	5.79 (7.53)	9.34 (8.00)	8.57 (7.53)	0.070	--								
BMI, kg/m^2^	26.07 (3.82)	28.23 (5.92)	28.18 (4.22)	21.69 (2.75)	<0.0001^a^	<0.0001^a^	30.65 (7.92)	36.51 (6.67)	34.33 (6.90)	21.54 (1.19)	<0.0001^a^	<0.0001^a^	<0.0001^a^	<0.0001^a^
Fasting glucose, mmol/l	11.64 (3.53)	5.89 (1.44)	7.98 (2.33)	6.33 (0.97)	<0.0001^a^	<0.0001^a^	11.61 (4.11)	6.63 (1.08)	7.76 (1.88)	6.66 (1.62)	<0.0001^a^	<0.0001^a^	0.92	0.19
HbA_1c_, mmol/mol	91.22 (16.11)	59.63 (13.21)	57.34 (12.57)	44.85 (12.98)	<0.0001^a^	<0.0001^a^	103.48 (17.35)	53.39 (13.88)	52.41 (14.12)	48.15 (9.04)	<0.0001^a^	<0.0001^a^	<0.0001^a^	<0.0001^a^
HbA_1c_, %	10.5 (3.6)	7.6 (3.4)	7.4 (3.3)	6.3 (3.3)	<0.0001^a^	<0.0001^a^	11.7 (3.7)	7.0 (3.4)	6.9 (3.4)	6.6 (3.0)	<0.0001^a^	<0.0001^a^	<0.0001^a^	<0.0001^a^
Fasting C-peptide, nmol/l	0.71 (0.41)	1.98 (0.63)	0.77 (0.42)	0.42 (0.20)	0.479	<0.0001^a^	0.90 (0.5)	2.43 (0.61)	1.19 (0.46)	0.76 (0.23)	<0.0001^a^	<0.0001^a^	0.0013^a^	<0.0001^a^
HOMA2-B	34.67 (21.47)	209.54 (41.32)	68.5 (39.13)	59.05 (19.27)	<0.0001^a^	<0.0001^a^	46.29 (35.19)	193.47 (37.16)	90.94 (35.22)	86.78 (30.71)	<0.0001^a^	<0.0001^a^	0.00052^a^	<0.0001^a^
HOMA2-IR	2.29 (1.49)	4.63 (1.71)	1.98 (1.12)	1.03 (0.47)	<0.0001^a^	<0.0001^a^	2.9 (1.78)	5.74 (1.39)	3.03 (1.30)	1.84 (0.61)	<0.0001^a^	<0.0001^a^	0.0043^a^	<0.0001^a^

Note: Values are mean (SD) unless otherwise indicated

^a^Bonferroni corrected significant *p* values

*p* value by ANOVA, *p*1 adjusted for duration of diabetes

In the sex-stratified analysis, SIDD (60.6%) remained the predominant cluster in men whereas the main subgroup was MOD (53.2%) in women; MARD was more common in men (13.3% vs 2.1%) (Fig. 1, Table 3).

**Fig. 2:**
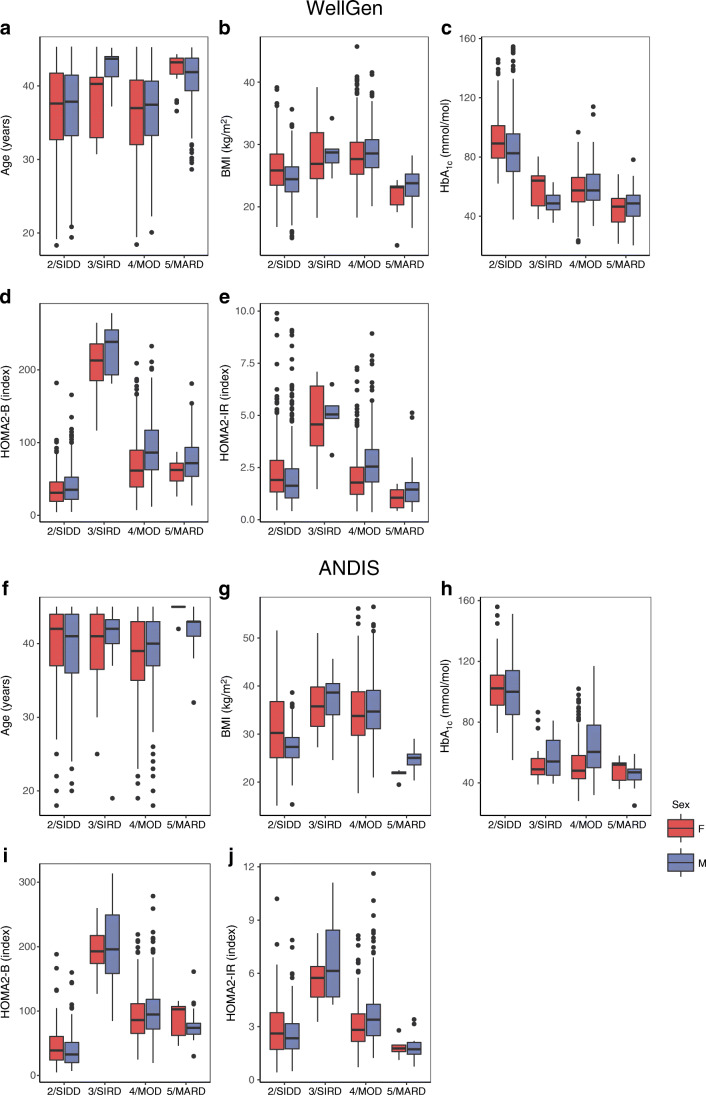
Box plot of cluster characteristics in the WellGen and ANDIS studies. Distribution of age at diagnosis (**a**, **f**), BMI (**b**, **g**), HbA_1c_ (**c**, **h**), HOMA2-B (**d**, **i**) and HOMA2-IR (**e**, **j**) in the WellGen (**a**–**e**) and ANDIS (**f**–**j**) studies for each cluster. The central line within each box represents the median and the upper and lower limits of the box represent the IQR. The whiskers are the most extreme values within 1.5× the IQR from the first and second quartiles. *k*-means clustering was done separately for men and women; data are shown for each sex separately. F, female sex; M, male sex

Concordant with the diabetes subgroups in ANDIS, among the two predominant subgroups, Indian individuals in the SIDD subgroup had the lowest insulin secretion (HOMA2-B) and the highest blood glucose levels while those in the MOD subgroup had the highest BMI. Individuals in the SIRD subgroup were the most insulin resistant, whereas those in the MARD subgroup were the oldest at diagnosis, with the lowest blood glucose levels and lowest degree of insulin resistance (Fig. 2, Table 3). These results support the pathophysiological basis for subclassification in a population which has a different genetic and socioeconomic background compared with the Swedish population.

The distribution of subgroups in ANDIS differed from that of WellGen; MOD was the most predominant cluster (67.57%), followed by SIDD (26.09%), SIRD (3.64%) and MARD (2.70%) (Fig. 1, Table 3). These distributions were similar in men and women. The pathophysiological characteristics of these Swedish individuals with young-onset type 2 diabetes were similar to those in the parent ANDIS cohort (Fig. 2, Table 3).

### Treatment

In both WellGen and ANDIS, insulin treatment (alone or in combination with OHAs) was most commonly prescribed to individuals in the SIDD subgroup (38.3% in WellGen, 51.0% in ANDIS) (Table 4).

**Table 4:** Treatment and complications by cluster in the WellGen and ANDIS study

Treatment/complication	WellGen (*N*=1612)							ANDIS (*N*=962)						
	SIDD	SIRD	MOD	MARD	*p* value	*p*1 value	*p*2 value	SIDD	SIRD	MOD	MARD	*p* value	*p*1 value	*p*2 value
No. of participants in subgroup	851	18	608	135				251	35	650	26			
Current treatment														
Diet only	68 (8.0)	4 (22.2)	54 (8.9)	9 (6.7)	0.146	0.544	0.922	4 (1.6)	5 (14.3)	60 (9.2)	8 (30.8)	0.0001^a^	0.0001^a^	0.002^a^
OHA only (SU + metformin ± thiazolidinedione)	457 (53.7)	13 (72.2)	409 (67.3)	100 (74.1)	0.0001^a^	0.0001^a^	0.0001^a^	117 (46.6)	33 (74.3)	470 (72.3)	16 (61.5)	0.0001^a^	0.0001^a^	0.0001^a^
Insulin only	58 (6.8)	0 (0)	19 (3.1)	8 (5.9)	0.013	0.002^a^	0.004^a^	36 (14.3)	0 (0)	23 (3.5)	0 (0)	0.0001^a^	0.0001^a^	0.0001^a^
Both OHA+ insulin^b^	268 (31.5)	1 (5.6)	126 (20.7)	18 (13.3)	0.0001^a^	0.0001^a^	0.0001^a^	92 (36.7)	4 (11.4)	96 (18.8)	2 (7.7)	0.0001^a^	0.0001^a^	0.0001^a^
Complications														
Cardiovascular disease (coronary events and/or stroke)^c^	61 (7.2)	0 (0)	39 (6.4)	20 (14.8)	0.004^a^	0.574	0.242	10 (4.0)	3 (8.6)	20 (3.1)	1 (3.8)	0.375	0.502	0.633
Coronary event	51 (6.0)	0 (0)	32 (5.3)	17 (12.6)	0.009	0.553	0.257	7 (2.8)	3 (8.6)	13 (2.0)	0 (0)	0.075	0.476	0.493
Stroke	13 (1.6)	0 (0)	9 (1.5)	4 (3.1)	0.582	0.942	0.746	3 (1.2)	0 (0)	7 (1.1)	1 (3.8)	0.522	0.884	0.980
Nephropathy (macroalbuminuria and/or CKD)^d^	340 (40.0)	6 (33.3)	147 (24.2)	68 (50.4)	0.0001^a^	0.0001^a^	0.0001^a^	76 (40.0)	19 (70.4)	206 (46.6)	9 (52.9)	0.022	0.126	0.087
Macroalbuminuria^e^	172 (21.1)	0 (0)	66 (11.3)	12 (9.4)	0.0001^a^	0.0001^a^	0.001^a^	25 (13.8)	5 (5.0)	59 (14.9)	1 (7.1)	0.484	0.731	0.635
CKD: eGFR^f,g^	247 (31.7)	6 (35.3)	98 (17.6)	62 (52.1)	0.0001^a^	0.0001^a^	0.0001^a^	62 (25.4)	16 (47.1)	165 (26.4)	8 (33.3)	0.049	0.765	0.475
Early CKD (60–90 ml min^−1^ [1.73m]^−2^)	190 (24.4)	6 (35.3)	85 (15.3)	49 (41.2)	0.0001^a^	0.0001^a^	0.006^a^	56 (23.5)	16 (47.1)	157 (25.4)	8 (33.3)	0.025	0.561	0.831
Moderate CKD (30–60 ml min^−1^ [1.73m]^−2^)	52 (6.7)	0 (0)	10 (1.8)	12 (10.1)	0.0001^a^	0.0001^a^	0.002^a^	5 (2.1)	0 (0)	6 (1.0)	0 (0)	0.471	0.196	0.162
Severe CKD (<30 ml min^−1^ [1.73m]^−2^)	5 (0.6)	0 (0)	3 (0.5)	1 (0.8)	0.966	0.811	0.898	1 (0.4)	0 (0)	2 (0.3)	0 (0)	0.970	0.893	0.935
Diabetic retinopathy (*n*=657)^h^	112 (36.8)	4 (57.1)	51 (18.0)	13 (21.0)	0.0001^a^	0.0001^a^	0.0001^a^	23 (24.7)	4 (44.4)	30 (16.6)	0 (0)	0.050	0.11	0.13
NPDR	105 (34.5)	4 (57.1)	46 (16.2)	12 (19.4)	0.0001^a^	0.0001^a^	0.0001^a^	22 (23.9)	5 (44.4)	29 (16.1)	0 (0)	0.050	0.050	0.225
PDR	7 (2.3)	0 (0)	5 (1.8)	1 (1.6)	0.936	0.642	0.847	1 (1.1)	0 (0)	1 (0.6)	0 (0)	0.948	0.948	0.821
Neuropathy^i^	322 (38.8)	9 (50.0)	323 (53.8)	56 (43.4)	0.0001^a^	0.0001^a^	0.003^a^	12 (4.8)	1 (2.9)	3 (0.5)	0 (0)	0.0001^a^	0.0001^a^	0.002^a^

Values are *n* (%)

^a^Bonferroni corrected significant *p* values

^b^Data for metformin + insulin only for ANDIS clusters

^c^CVD: data available for all (*n*=1612) for WellGen, *n*=956 (SIDD *n*=250, SIRD *n*=35, MOD *n*=645, MARD *n*=26) for ANDIS

^d^Nephropathy: data available for all (*n*=1612) for WellGen, *n*=676 (SIDD *n*=190, SIRD *n*=27, MOD *n*=442, MARD *n*=17) for ANDIS

^e^Macroalbuminuria: data available for *n*=1544 (SIDD *n*=816, SIRD *n*=18, MOD *n*=582, MARD *n*=128) for WellGen, *n*=611 (SIDD *n*=181, SIRD *n*=20, MOD *n*=396, MARD *n*=14) for ANDIS

^f^Based on MDRD formula

^g^CKD: eGFR: data available for *n*=1471 (SIDD *n*=779, SIRD *n*=17, MOD *n*=556, MARD *n*=119) for WellGen, *n*=927 (SIDD *n*=244, SIRD *n*=34, MOD *n*=625, MARD *n*=24) for ANDIS

^h^Diabetic retinopathy: data available for *n*=657 (SIDD *n*=304, SIRD *n*=7, MOD *n*=284, MARD *n*=62) for WellGen and *n*=288 (SIDD *n*=92, SIRD *n*=9, MOD *n*=180, MARD *n*=7) for ANDIS

^i^Neuropathy: data available for *n*=1576 (SIDD *n*=829, SIRD *n*=18, MOD *n*=600, MARD *n*=129), diagnosed using Biothesiometry for WellGen, *n*=956 (SIDD *n*=250, SIRD *n*=35, MOD *n*=645, MARD *n*=26), ICD codes ICD-10 = E104 or E114 for ANDIS

*p* value was calculated by χ^2^ test and *p*1 value by χ^2^ test for SIDD and MOD clusters only; *p*2 value was calculated by logistic regression, adjusted for duration of diabetes and sex for SIDD and MOD clusters

NPDR, non-proliferative diabetic retinopathy; OHA, oral glucose-lowering agent; PDR, proliferative diabetic retinopathy; SU, sulfonylurea

### Complications

In WellGen, we compared the prevalence of complications in the two major subtypes, SIDD and MOD. Small numbers in the SIRD and MARD groups precluded comparison of complications. Retinopathy and nephropathy were most common in the SIDD subgroup whereas neuropathy was more prevalent in MOD (Table 4). The prevalence of macrovascular complications was similar in these two subtypes. Of the less common subgroups, SIRD had a high prevalence of retinopathy while MARD had a high prevalence of nephropathy and macrovascular disease.

In the ANDIS cohort, nephropathy (70.4%) and retinopathy (44.4%) prevalence were highest in SIRD whereas neuropathy was most common in SIDD (Table 4). Consistent with previous findings, SIRD also showed the highest prevalence of CKD (47.1%) while macroalbuminuria (14.9%) was most common in MOD.

### De novo clusters show a high degree of concordance with SIDD and MOD

We also applied the de novo *k*-means clustering to assess the subgroups obtained in the Indian study population and compared them with those obtained using the previously published algorithm. Two was the optimum number of clusters (ESM Fig. 2a); cluster 1 had a prevalence of 66.6% while cluster 2 had a prevalence of 33.4% (ESM Table 7). Cluster 1 showed 88.8% concordance with SIDD (86.8% in male participants, 92.4% in female participants) while cluster 2 had an overlap of 62.5% with MOD (83.9% in male participants, ~49.5% in female participants) (ESM Fig. 2b).

Both clusters had the same cluster characteristics as seen using the centroid method, thereby providing a technical replication (ESM Table 7). The similarity also extended to complication rates, with nephropathy and retinopathy being prevalent in cluster 1 compared with cluster 2 whereas neuropathy was more prevalent in cluster 2 (ESM Table 8).

### Low prevalence of genetic type 1 diabetes in WellGen type 2 diabetes subgroups

In the absence of GAD autoantibody data, a previously established type 1 diabetes GRS [25] comprising nine SNPs (ESM Table 4), which was validated in the Indian population [23], was applied to estimate the proportion of participants with autoimmune diabetes in a subset of the WellGen cohort (ESM Table 5). The GRS was associated with the positive control participants with type 1 diabetes compared with SIDD and MOD. The same GRS did not associate with either SIDD or MOD compared with non-diabetic control individuals (ESM Fig. 3, ESM Table 9). The proportion of participants with GRS ≥90% and 80% was ~5% and 28.7%, respectively, in type 1 diabetes (positive controls) whereas it was 0% and 1.4% in SIDD, 0% and 4.7% in MOD, and 0% and 1% in control participants. The same GRS was associated with SAID in young ANDIS participants (β = 7.3 ± 0.72, *p* < 2 × 10^−16^).

### Indian diabetes subgroups are similar to subgroups in European cohort DIREVA with longer diabetes duration

Given the difference in duration of diabetes in ANDIS and WellGen, we compared the WellGen subgroups with those in the Finnish cohort DIREVA (*n* = 420) with duration of diabetes 14.4 years (ESM Table 10). The differences in proportion of subgroups and cluster characteristics between WellGen and DIREVA were similar to those between WellGen and ANDIS. Similar to ANDIS, SIDD was less common compared with MOD in DIREVA (23.6% vs 70.8%) (ESM Table 10, ESM Fig. 4).

### Subgroups of diabetes in other regions of India

We applied the Swedish algorithm [2] to two studies from different geographical regions in India, Ahmedabad, Gujarat, western India (*N* = 187) and Dibrugarh, Assam, north-eastern India (*N* = 205; PHENOEINDY-2 cohort) (ESM Tables 11, 12). Concordant with findings in WellGen, the Ahmedabad cohort had the highest proportion of participants in the SIDD subgroup (56.68%) followed by MOD (33.15%) and MARD (10.16%) (ESM Table 11). The similarity extended to the subgroup distributions in the two sexes: the SIDD subgroup was most prevalent in male participants (61.59%) while MOD was most prevalent in female participants (53.06%) (ESM Table 11, ESM Fig. 5). The PHENOEINDY-2 cohort was the youngest and the thinnest cohort of all; the proportion of SIDD (66.66%) was the highest, followed by MOD (23.20%), MARD (7.72%) and SIRD (1.40%) (ESM Table 12, ESM Fig. 6).

## Discussion

We showed that the clusters described in the newly diagnosed unselected European type 2 diabetes patients [2, 3] are also seen in the younger and thinner Indians. SIDD and MOD were the two predominant subgroups, while MARD was less common and SIRD the least common in both populations. SIDD was the predominant cluster in Indians whereas MOD was the most prevalent in Europeans. The predominance of SIDD was replicated in two independent, geographically distinct Indian cohorts of individuals with young-onset type 2 diabetes.

The distribution of the clusters suggests that deficient insulin secretion, rather than the often-purported insulin resistance, is the driver of young-onset type 2 diabetes in India. In contrast, in the young Swedish and Finnish type 2 diabetes populations, obesity and insulin resistance seemed to be the primary pathophysiological drivers. The proposed prominent role of insulin resistance was based on previous demonstrations of higher insulin resistance in Indians compared with Europeans at a given BMI possibly due to relatively more adipose body composition [7, 28, 29]. Despite the differences in age, BMI and duration of diabetes, the characteristics of the clusters themselves in Indians broadly reflected those in the European studies [2] Our new findings suggest a paradigm shift for the understanding of the pathophysiology of type 2 diabetes in young Indians, albeit they do not preclude the role of insulin resistance.

De novo *k*-means subclassification validated the two major diabetes subgroups obtained from the European-derived centroids. The concordance was greater in men for both subgroups, while it was lower in women for the newly obtained cluster 2 with MOD. While this increases our confidence in the classification, reclassification of a proportion of women with MOD to a SIDD equivalent cluster 1 highlights the role of insulin deficiency in the pathogenesis of type 2 diabetes in young Indians.

We applied the European-derived centroids to two smaller cohorts of individuals with young-onset type 2 diabetes from western (Ahmedabad, Gujarat) and north-eastern (Dibrugarh, Assam) India. The proportion of subclasses in the Ahmedabad participants was nearly identical to those in Pune, while the proportion of individuals in the SIDD subgroup was highest in Dibrugarh. Gujarat is a more affluent state while Assam has a lower development index and high prevalence of undernutrition.

Physicians in India have long realised the phenotypic differences of Indian diabetes patients compared with those described in patients of European origin [30, 31]. Interestingly, lean type 2 diabetes has been prominently reported in the impoverished states of Orissa and north-east India, where malnutrition-related diabetes (MRDM) has been described [32]. A proportion of SIDD patients from Assam could well be characterised similarly. There is an increasing recognition that early-life undernutrition could lead to smaller beta cell mass and insulin secretion defects demonstrable from early childhood in serially studied birth cohorts and could manifest as prediabetes or type 2 diabetes in young adulthood [33, 34]. Animal studies have clearly demonstrated poor beta cell development and islet dysfunction in offspring born to malnourished pregnant mothers [35–37]. It is intriguing that the highest rise in the prevalence of type 2 diabetes in India over the last 25 years has been demonstrated in states that have suffered chronic environmental, socioeconomic and nutritional deficits [38]. On such a background of intergenerational deprivation, a relatively small socioeconomic development appears enough to precipitate diabetes at a young age. It is of note that the prevalence of diabetes in those above 20 years of age has increased from 5.5% to 7.5% between 1990 and 2016 in the state of Assam.

Individuals in the diabetes subgroups displayed different sensitivities to micro- and macrovascular complications. Microvascular disorders of the retina and kidney were more prevalent in SIDD compared with MOD, while peripheral nerve damage was more prevalent in MOD. Possible reasons for these differences may lie in the pathophysiological mechanisms driving the subgroups and this needs to be studied further. Prevalence of macrovascular disease was similar in two subgroups. In the original Swedish classification, the SIRD subgroup generated a lot of interest given the high propensity of affected individuals for developing nephropathy. SIRD was the smallest subgroup in Indians with high insulin resistance as well as insulin secretion, although it was heterogeneous between the Indian cohorts. Intriguingly, the MARD subgroup had a strikingly high rate of macrovascular disease. The unique profiles of these subgroups could well represent population-specific differences and highlight the need for customisation of the clustering algorithm.

Other studies have investigated the heterogeneity of type 2 diabetes in Indians. The INSPIRED study from a chain of private diabetes clinics in India reported four subclasses, two of which were similar to the Swedish study (SIDD and MARD) [5] and two of which were new (insulin-resistant obese diabetes [IROD] and combined insulin resistance and deficient diabetes [CIRDD]) [14]. However, the clustering parameters were different and therefore not directly comparable with our study. The MASALA-MESA study reported subclasses in a mixed population in the USA, including migrant South Asian Indians (*n* = 217) [15]. They found an excess of younger, thinner and severe hyperglycaemic individuals among South Asian Indians, supporting our findings.

This study has strengths and limitations. This is the first attempt in India to subclassify patients with a diagnosis of type 2 diabetes at a young age. The presence of subgroups in our study comparable with those in a genetically and historically distinct European population validates the subgroups. While the power of the study is limited, validation by de novo clustering increases confidence in classification. The Indian patients are clinic-based and enrolled many years after diabetes diagnosis while on glucose-lowering treatment, which may affect the proportions of subclasses. We cannot rule out the possibility that some individuals might shift to different subgroups over time; however, only a small proportion did so in another study [3]. A sensitivity analysis in WellGen showed that the proportions of the subgroups varied with increasing diabetes duration, although SIDD remained the predominant subgroup (45.5%) even in those with less than 5 years of diagnosis. Observations in other two Indian cohorts further validated these findings. Another limitation is that this study is an opportunistic comparison of existing data and therefore laboratory measurements are not fully harmonised between cohorts. However, C-peptide measurements in different cohorts were calibrated against the same WHO standard, facilitating comparisons. Given the lack of GAD autoantibody data, it might be suspected that the SIDD group in WellGen includes individuals with autoimmune diabetes (latent autoimmune diabetes in adults [LADA]). However, the low prevalence of individuals with high type 1 diabetes GRS scores in WellGen deems a large contribution of autoimmune diabetes extremely unlikely.

In summary, we demonstrate the applicability of a European algorithm for subclassifying type 2 diabetes in young Indian patients. Our results demonstrate a prominent role for insulin secretion defects in the pathophysiology of diabetes in this group. These results could potentially influence treatment strategies for achieving optimal metabolic control, with possible benefits for long-term health [4, 39]. Translation to personalised medicine will come from carefully designed prospective studies including genetic and epigenetic investigations to elucidate pathophysiological mechanisms underlying the subgroups.

## Supplementary information

ESM (PDF 837 kb)

## Data Availability

The datasets generated during and/or analysed during the current study are available from the corresponding author on reasonable request.

## Abbreviations

CKD: Chronic kidney disease
GRS: Genetic risk score
MARD: Mild age-related diabetes
MDRD: Modification of Diet in Renal Disease
MOD: Mild obesity-related diabetes
SAID: Severe autoimmune diabetes
SIDD: Severe insulin-deficient diabetes
SIRD: Severe insulin-resistant diabetes
