# Supplementary material for: Subgroups of patients with young-onset type 2 diabetes in India reveal insulin deficiency as a major driver
Source: Diabetologia. 2021 Oct 23;65(1):65–78. doi: 10.1007/s00125-021-05543-y (PMC8660725; doi:10.1007/s00125-021-05543-y)
Supplement: Supplementary file 1 — (PDF 837 kb) [file 125_2021_5543_MOESM1_ESM.pdf]

Subgroups of young-onset type 2 diabetes in India reveal insulin deficiency as a major driver

Authors: Rashmi B Prasad, Olof Asplund, Sharvari R Shukla, Rucha Wagh, Pooja Kunte, Dattatrey Bhat, Malay Parikh, Meet Shah, Sanat Phatak, Annemari Käräjämäki, Anupam Datta, Sanjeeb Kakati, Tiinamaija Tuomi, Banshi Saboo, Emma Ahlqvist, Leif Groop, Chittaranjan S Yajnik

ESM Methods, Tables and Figures

ESM Methods

Comparability of laboratory measurements

C peptide was measured by ELISA (DBC) in the WellGen and Assam study, and by ECLIA (Roche) in the ANDIS and DIREVA study and by CLIA (MAGLUMI) in Ahmedabad study; all based on mouse anti-C-peptide antibody and calibrated against WHO International Reference Reagent for C-peptide of human insulin for immunoassay (IRR code 84/510). This highlights comparability of the assays.

GWAS QC and Imputation

SNP exclusion criteria included missingness threshold of > 0.05% MAF<1% and Hardy-Weinberg equilibrium p-value<0.05. Imputation was performed on the Michigan Imputation server using 1000G Phase 3 v5 (GRCh37/hg1) as a reference panel along with Eagle v2.4 phasing and SAS (South Asian) as population type.

ESM Tables

	Ahmedabad		
**Characteristics**	**Men**	**Women**	**All**
Number	138 (73.79)	49 (26.20)	187
Age at Diagnosis (y)	36.6 (6.75)	37.47 (6.72)	36.83 (6.74)
BMI (Kg/m^2)	27.44 (3.7)	28.28 (4.16)	27.66 (3.83)
Fasting glucose (mmol/L)	10.70 (4.64)	11.37 (4.78)	10.87 (4.68)
HbA1c (mmol)	72.61 (26.2)	72.91 (25)	72.69 (25.82)
HbA1c (%)	8.8 (4.5)	8.8 (4.4)	8.8 (4.5)
Fasting C-peptide (nmol/L)	0.45 (0.21)	0.41 (0.16)	0.44 (0.20)
HOMA2-B	37.41 (31.54)	30.35 (23.94)	35.56 (29.84)
HOMA2-IR	1.5 (1.1)	1.48 (1.01)	1.49 (1.07)

ESM table 1: Clinical and Biochemical characteristics of participants enrolled in Ahmedabad study with age at diagnosis less than 45 years

	Assam (n=205)		
**Characteristics**	**Men**	**Women**	**All**
Number	135 (65.85)	70 (34.15)	205
Age at Diagnosis (y)	32.93 (4.86)	30.99 (5.8)	32.26 (5.27)
Duration of Diabetes (y)	2.87 (3.38)	4.33 (3.85)	3.37 (3.60)
BMI (Kg/m^2)	23.46 (3.86)	23.21 (3.65)	23.37 (3.79)
Fasting glucose (mmol/L)	10.32 (4.85)	10.90 (4.93)	10.52 (4.87)
HbA1c (mmol)	82.3 (33.38)	82.15 (30.98)	82.25 (32.5)
HbA1c (%)	9.7 (5.2)	9.7 (5.0)	9.7 (5.1)
Fasting C-peptide (nmol/L)	0.40 (0.31)	0.45 (0.38)	0.42 (0.34)
HOMA2-B	38.96 (35.35)	34.93 (28.45)	37.58 (33.14)
HOMA2-IR	1.55 (2.71)	2.26 (4.98)	1.79 (3.65)

ESM table 2: Clinical and Biochemical characteristics of participants enrolled in **PHENOEINDY-2** study from Assam with age at diagnosis less than 45years

	DIREVA		
**Characteristics**	**Men**	**Women**	**All**
Number	243	177	420
Age at Diagnosis (y)	36.78 (7.23)	36.44 (6.95)	36.6 (7.1)
Duration of Diabetes (y)	14.78 (12.06)	13.85 (12.30)	14.39 (12.16)
BMI (Kg/m^2)	31.62 (6.59)	31.63 (6.60)	31.62 (6.6)
Fasting glucose (mmol/L)	9.19 (3.06)	8.74 (3.25)	9.01 (3.15)
HbA1c (mmol/mol)	59.32 (16.93)	56.48 (16.84)	58.13 (16.93)
HbA1c (%)	7.5 (3.7)	7.3 (3.7)	7.5 (3.7)
Fasting C-peptide (nmol/L)	0.60 (0.51)	0.59 (0.41)	0.59 (0.47)
HOMA2-B	46.80 (36.26)	53.61 (36.17)	49.67 (34.09)
HOMA2-IR	1.73 (1.36)	1.65 (1.03)	1.69 (1.23)

Note: Values are mean (SD),

ESM table 3: Clinical and Biochemical characteristics of participants enrolled in DIREVA study with age at diagnosis less than 45years.

Sl No	SNP	Gene	Major allele	Minor allele	Risk allele	Odds Ratio	Weight
1	rs2187668	HLA-DRB1	C	T	T	-	-
2	rs7454108	HLA-DQ8	C	T	C	-	-
	rs3957146		C	T	C	-	-
3	rs1264813	HLA_A_24	C	T	T	1.54	0.43
4	rs2395029	HLA_B_5701	T	G	T	2.5	0.92
5	rs3129889	HLA_DRB1_15	G	A	A	14.88	2.7
6	rs2476601	PTPN22	A	G	A	1.96	0.67
7	rs689	INS	A	T	T	1.75	0.56
8	rs12722495	IL2RA	T	C	T	1.58	0.46
9	rs2292239	ERBB3	G	T	T	1.35	0.3

SNPs	Haplotypes	Haplotypes	Haplotype allele score	Odds ratio	Weights
rs2187668, rs7454108* (proxy 1: rs3957146)	DR3/DR4	CT/CT	6	48.18	3.87
	DR4/DR4	CC/CC	5	21.98	3.09
	DR3/DR3	TT/TT	4	21.12	3.05
	DR4/X	CC/CT	3	7.03	1.95
	DR3/X	CT/TT	2	4.53	1.51
	X/X	CC/TT	1	1	0

*Barker et.al., Winkler et.al.

ESM table 4. List of type 1 diabetes associated SNPs used to construct genetic risk scores for T1D presented with the risk alleles.

	Included (N=560)	Excluded (N=1052)	P-value
Age at Diagnosis (y)	37.212 (5.832)	36.863 (5.749)	0.25
BMI (Kg/m^2)	26.371 (4.028)	26.079 (4.09)	0.167
Fasting glucose (mmol/l)	9.278 (3.265)	9.251 (3.38)	0.879
HbA1c (mmol/mol)	71.271 (22.532)	73.185 (22.737)	0.106
HbA1c (%)	8.3 (4.2)	8.8 (4.3)	0.106
Fasting C-Peptide (nmol/l)	0.748 (0.51)	0.779 (0.434)	0.213
HOMA2B	54.428 (37.141)	59.419 (42.854)	0.015
HOMA2IR	2.102 (1.558)	2.144 (1.257)	0.58

Values are mean (SD)
[a]Bonferroni corrected significant *p* values
p values were calculated using *t* test

ESM table 5: Patient characteristics for those included in the T1D GRS calculation compared to those excluded.

	WellGen-Total (1612)											
	Duration of diabetes < 5 y (n=580, 36.0%)				Duration of diabetes ≥ 5y (n=1032, 64.0%)							
**Cluster**	**SIDD**	**SIRD**	**MOD**	**MARD**	**SIDD**	**SIRD**	**MOD**	**MARD**	**P (SIDD)**	**P (SIRD)**	**P (MOD)**	**P (MARD)**
**Number (%)**	264 (45.5)	10 (1.7)	259 (44.7)	47 (8.1)	587 (56.9)	8 (0.8)	349 (33.8)	88 (8.5)				
Age at Diagnosis (y)	37.1 (6.0)	37.0 (5.4)	37.0 (5.)	41.3 (3.9)	36.8 (5.7)	41.0 (3.4)	35.7 (5.7)	40.6 (4.0)	0.588	0.095	0.006[a]	0.346
Duration of diabetes (y)	1.9 (1.5)	1.5 (1.7)	1.9 (1.5)	2.1 (1.4)	14.3 (7.0)	12.6 (6.6)	13.7 (7.2)	13.9 (7.7)	--	--	--	--
BMI (Kg/m^2)	24.7 (3.5)	29.3 (6.3)	28.4 (4.1)	23.9 (2.3)	25.1 (3.4)	28.4 (5.3)	28.4 (3.9)	22.8 (2.4)	0.074	0.419	0.804	0.013
Fasting glucose (mmol/L)	10.4 (3.0)	5.7 (0.79)	7.4 (1.9)	6.7 (1.5)	10.9 (3.5)	6.0 (1.8)	8.2 (2.6)	6.4 (1.4)	0.047	0.700	0.0001[a]	0.188
HbA1c (mmol/mol)	88.1 (21.0)	53.4 (11.9)	57.1 (12.9)	46.1 (10.6)	86.5 (18.5)	60.9 (14.1)	59.2 (12.8)	47.9 (10.3)	0.257	0.247	0.054	0.338
HbA1c (%)	10.2 (4.1)	7.0 (3.2)	7.4 (3.3)	6.4 (3.2)	10.1 (3.9)	7.7 (3.4)	7.5 (3.3)	6.5 (3.2)	0.257	0.247	0.054	0.338
Fasting C-Peptide (nmol/L)	0.75 (0.40)	2.1 (0.47)	0.96 (0.46)	0.67 (0.29)	0.65 (0.39)	2.0 (0.72)	0.84 (0.49)	0.54 (0.28)	0.001	0.856	0.004[a]	0.014
HOMA2B	42.2 (24.4)	218.9 (32.7)	85.8 (41.7)	77.1 (33.6)	35.6 (20.9)	210.1 (51.57)	71.3 (41.2)	71.9 (29.8)	0.0001	0.665	0.0001[a]	0.361
HOMA2IR	2.2 (1.3)	4.7 (1.2)	2.4 (1.2)	1.6 (0.79)	2.03 (1.47)	4.7 (2.0)	2.19 (1.31)	1.31 (0.73)	0.169	0.992	0.048	0.016

	WellGen-Male participants (902)											
	Duration of diabetes < 5 y (N=342, 37.9%)				Duration of diabetes ≥ 5y (N=560, 62.1%)							
**Cluster**	**SIDD**	**SIRD**	**MOD**	**MARD**	**SIDD**	**SIRD**	**MOD**	**MARD**	**P (SIDD)**	**P (SIRD)**	**P (MOD)**	**P (MARD)**
**Number (%)**	180 (52.6)	2 (0.6)	120 (35.1)	40 (11.7)	367 (65.5)	3 (0.5)	110 (19.6)	80 (14.3)				
Age at Diagnosis (y)	37.1 (5.9)	43.8 (0.21)	36.9 (5.5)	41.0 (4.1)	36.9 (5.5)	41.2 (3.9)	36.2 (5.3)	40.5 (4.1)	0.726	0.440	0.281	0.570
Duration of diabetes (y)	1.9 (1.5)	3.5 (0.27)	1.9 (1.5)	2.06 (1.3)	14.8 (7.5)	11.06 (7.2)	14.0 (7.4)	13.9 (7.9)	--	--	--	--
BMI (Kg/m^2)	24.2 (3.2)	27.9 (1.6)	28.1 (3.1)	24.1 (2.3)	24.5 (3.0)	29.3 (4.8)	29.5 (4.1)	23.0 (2.3)	0.208	0.715	**0.003**	0.015
Fasting glucose (mmol/L)	10.1 (2.9)	5.9 (0.71)	7.3 (2.1)	6.7 (1.6)	10.4 (3.4)	5.6 (1.3)	7.9 (2.7)	6.4 (1.6)	0.331	0.792	0.073	0.252
HbA1c (mmol/mol)	87.9 (23.5)	53.5 (13.1)	56.7 (11.8)	46.9 (9.2)	83.0 (18.8)	46.1 (9.5)	63.4 (14.2)	47.9 (10.4)	0.008	0.507	**0.0001**	0.609
HbA1c (%)	10.2 (4.3)	7.0 (3.3)	7.5 (3.2)	6.4 (3.0)	9.7 (3.9)	6.4 (3.0)	7.9 (3.4)	6.5 (3.1)	0.008	0.507	**0.0001**	0.609
Fasting C-Peptide (nmol/L)	0.73 (0.41)	2.28 (0.04)	1.05 (0.45)	0.70 (0.31)	0.63 (0.37)	2.10 (0.62)	1.12 (0.57)	0.56 (0.29)	0.002	0.723	0.290	0.014

HOMA2B	43.7 (25.1)	224.0 (43.7)	94.4 (42.2)	80.1 (34.9)	37.2 (20.8)	232.6 (48.6)	89.9 (42.9)	73.3 (30.2)	0.001	0.854	0.433	0.278
HOMA2IR	2.06 (1.20)	5.26 (0.29)	2.62 (1.27)	1.71 (0.81)	1.91 (1.41)	4.81 (1.69)	2.90 (1.55)	1.71 (0.81)	0.210	0.751	0.133	0.015
	**WellGen-Female participants (710)**											
	**Duration of diabetes < 5 y (N=238, 33.5%)**				**Duration of diabetes ≥ 5y (N=472, 66.5%)**							
**Cluster**	**SIDD**	**SIRD**	**MOD**	**MARD**	**SIDD**	**SIRD**	**MOD**	**MARD**	**P (SIDD)**	**P (SIRD)**	**P (MOD)**	**P (MARD)**
**Number (%)**	84 (35.3)	8 (3.4)	139 (58.4)	7 (2.9)	220 (46.6)	5 (1.1)	239 (50.6)	8 (1.7)				
Age at Diagnosis (y)	36.9 (6.3)	35.4 (4.6)	37.0 (5.9)	42.9 (1.1)	36.6 (5.9)	40.9 (3.6)	35.5 (5.9)	41.2 (3.2)	0.709	0.045	0.013	0.194
Duration of diabetes (y)	1.9 (1.5)	0.99 (1.5)	2.0 (1.5)	2.4 (1.9)	13.6 (6.0)	13.4 (6.8)	13.6 (7.1)	13.9 (6.3)	--	--	--	--
BMI (Kg/m^2)	25.8 (3.8)	29.6 (7.1)	28.6 (4.8)	22.7 (1.4)	26.2 (3.8)	25.9 (2.7)	27.9 (3.8)	20.7 (3.4)	0.428	0.289	0.152	0.172
Fasting glucose (mmol/L)	11.1 (3.2)	5.7 (0.85)	7.5 (1.8)	6.6 (1.1)	11.8 (3.6)	6.2 (2.2)	8.2 (2.5)	6.1 (0.9)	0.122	0.551	0.003[a]	0.292
HbA1c (mmol/mol)	88.5 (14.5)	53.4 (12.6)	57.5 (13.9)	41.4 (16.7)	92.3 (16.6)	69.6 (6.6)	57.2 (11.7)	47.8 (8.7)	0.067	0.023	0.859	0.363
HbA1c (%)	10.2 (3.5)	7.0 (3.3)	7.4 (3.4)	5.9 (3.7)	10.6 (3.7)	8.5 (2.8)	7.4 (3.2)	6.5 (3.0)	0.067	0.023	0.859	0.363
Fasting C-Peptide (nmol/L)	0.79 (0.38)	2.0 (0.52)	0.88 (0.46)	0.49 (0.19)	0.69 (0.42)	1.95 (0.84)	0.72 (0.39)	0.35 (0.21)	0.051	0.888	0.0001[a]	0.188
HOMA2B	39.2 (22.9)	217.6 (33.1)	78.5 (39.9)	60.2 (17.9)	32.9 (20.7)	196.6 (53.5)	62.7 (37.5)	58.0 (21.5)	0.023	0.395	0.001[a]	0.837
HOMA2IR	2.4 (1.3)	4.6 (1.3)	2.2 (1.2)	1.2 (0.49)	2.24 (1.54)	4.7 (2.3)	1.8 (1.0)	0.88 (0.43)	0.343	0.947	0.004[a]	0.192
HOMA2B	42.2 (24.4)	218.9 (32.7)	85.8 (41.7)	77.1 (33.6)	35.6 (20.9)	210.1 (51.57)	71.3 (41.2)	71.9 (29.8)	0.0001	0.665	0.0001[a]	0.361
HOMA2IR	2.2 (1.3)	4.7 (1.2)	2.4 (1.2)	1.6 (0.79)	2.03 (1.47)	4.7 (2.0)	2.19 (1.31)	1.31 (0.73)	0.169	0.992	0.048	0.016

Note: Values are mean (SD),
[a]Bonferroni corrected significant *p* values
p value by ANOVA,

ESM table 6: Sensitivity Analysis. Characteristics of participants enrolled in WellGen study by duration of diabetes and clusters, presented separately for men and women.

	Male participants (902)				Female participants (710)				Total (1612)			
**k-means**	**1**	**2**	**p**	**p1**	**1**	**2**	**p**	**p1**	**1**	**2**	**p**	**p1**
**Number (%)**	590 (65.4)	312 (34.6)			483 (68.0)	227 (32.0)			1073 (66.6)	539 (33.4)		
Age at Diagnosis (y)	36.66 (5.76)	38.76 (4.92)	<0.0001[a]	<0.0001[a]	35.94 (6.02)	37.59 (5.77)	<0.0001[a]	<0.0001[a]	36.033 (5.89)	38.27 (5.14)	<0.0001[a]	<0.0001[a]
Duration of diabetes (y)	10.33 (8.64)	8.48 (8.21)	0.0001[a]	--	10.40 (7.53)	8.00 (7.69)	0.0001[a]	--	10.36 (8.16)	8.28 (7.16)	0.0001[a]	--
BMI (Kg/m^2)	24.04 (2.94)	28.05 (3.69)	<0.0001[a]	<0.0001[a]	25.99 (3.60)	29.57 (4.54)	<0.0001[a]	<0.0001[a]	24.92 (3.40)	28.69 (4.14)	<0.0001[a]	<0.0001[a]
Fasting glucose (mmol/l)	9.51 (3.18)	8.28 (3.19)	<0.0001[a]	<0.0001[a]	10.37 (3.43)	7.57 (2.64)	<0.0001[a]	<0.0001[a]	9.90 (3.32)	7.98 (2.99)	<0.0001[a]	<0.0001[a]
HbA1c (mmol/mol)	77.41 (23.27)	65.30 (20.27)	<0.0001[a]	<0.0001[a]	78.09 (21.31)	57.86 (17.20)	<0.0001[a]	<0.0001[a]	77.72 (22.41)	62.17 (19.45)	<0.0001[a]	<0.0001[a]
HbA1c (%)	9.2 (4.3)	8.1 (4.0)	<0.0001[a]	<0.0001[a]	9.3 (4.1)	7.4 (3.7)	<0.0001[a]	<0.0001[a]	9.3 (4.2)	7.8 (3.9)	<0.0001[a]	<0.0001[a]
Fasting C-Peptide (nmol/l)	0.55 (0.26)	1.19 (0.48)	<0.0001[a]	<0.0001[a]	0.59 (0.30)	1.13 (0.51)	<0.0001[a]	<0.0001[a]	0.57 (0.28)	1.16 (0.49)	<0.0001[a]	<0.0001[a]
HOMA2B	40.87 (22.91)	92.42 (44.29)	<0.0001[a]	<0.0001[a]	36.94 (19.99)	97.77 (46.21)	<0.0001[a]	<0.0001[a]	39.10 (21.72)	94.67 (45.14)	<0.0001[a]	<0.0001[a]
HOMA2IR	1.54 (0.80)	3.19 (1.60)	<0.0001[a]	<0.0001[a]	1.78 (1.02)	2.91 (1.61)	0.50	0.73	1.65 (0.91)	3.07 (1.61)	<0.0001[a]	<0.0001[a]

Note: Values are mean (SD), p-value by ANOVA, p1 adjusted for duration of diabetes. Bonferroni corrected significant p-values are indicated with a.

ESM table 7: Characteristics of participants enrolled in WellGen study by clusters generated using *de novo* k-means clustering, presented separately for men and women.

	WellGen (N=1612)			
	**1**	**2**	**p-value**	**p1 value**
**Number (%)**	1073 (66.6)	539 (33.4)		
**Current Treatment**				
Only on Diet	68 (6.3)	67 (12.4)	0.0001[a]	0.0001[a]
Only on OHA (SUs+Metofrmin+/-Glitazones)	605 (56.4)	374 (69.4)		
Only on Insulin	72 (6.7)	13 (2.4)		
Both OHA+ Insulin	328 (30.6)	85 (15.8)		
Complications				
**Cardiovascular disease**	**76 (7.1)**	**44 (8.2)**	0.436	0.050
Coronary Events	63 (5.9)	37 (6.9)		
Stroke	17 (1.6)	9 (1.7)		
**Nephropathy (Macroalbuminuria and / or CKD) (n=1612)**	**406 (37.8)**	**155 (28.8)**	0.0001[a]	0.076
**Macroalbuminuria** [b]	165 (16.0)	85 (16.5)	0.813	0.271
**CKD: e-Glomerular Filtration Rate[c, d] (n=1471)**	**316 (32.7)**	**97 (19.2)**	0.0001[a]	0.0001[a]
Early CKD (60-90)	249 (25.8)	81 (16.0)		
Moderate (30-60)	62 (6.4)	12 (2.4)		
Severe (<30)	5 (0.6)	4 (0.8)		
**Diabetic Retinopathy (n=657)** [e]	**144 (32.1) (n=448)**	**36 (17.2) (n=209)**	0.0001[a]	0.013
NPDR	135 (30.1)	32 (15.3)		
PDR	9 (2.0)	4 (1.9)		
**Neuropathy** [f]	**449 (43.0)**	**261 (49.2)**	0.020	0.697

Values are number (%). p-value by Chi-square test.
a Bonferroni corrected significant p-values
b N=1020 for WellGen.
c Based on MDRD formula.
d N=1039 for WellGen.
e N=441 for WellGen.
f N=1031, diagnosed using biothesiometry for WellGen

ESM table 8: Treatment and complications by cluster in the WellGen by clusters generated using *de novo* k-means clustering.

	Beta	SE	Z	P
**WellGen**				
**T1D vs controls (positive control)**	15.43	1.24	12.47	<2e-16[a]
**SIDD vs controls**	-1.38	1.07	-1.29	0.197
**MOD vs controls**	-0.90	1.08	-0.83	0.40
**T1D vs SIDD**	14.30	1.33	10.73	<2e-16[a]
**T1D vs MOD**	13.01	1.31	9.93	<2e-16[a]

Bonferroni corrected significant p-values are indicated with a.

ESM table 9. Association of type 1 diabetes genetic risk scores with T1D (as positive control), SIDD, and MOD clusters.

	DIREVA						WellGen vs DIREVA	
	All (420)							
**Cluster**	**SIDD**	**SIRD**	**MOD**	**MARD**	**p**	**p1**	**p: SIDD**	**p:MOD**
**Number (%)**	99 (23.57)	1 (3.0)	297 (70.7)	23 (5.47)				
Age at Diagnosis (y)	34.30 (7.70)	41.20 (0)	36.99 (6.88)	41.94 (2.16)	< 0.0001[a]	< 0.0001[a]	<0.0001[a]	0.08
Duration of diabetes (y)	20.48 (13.26)	0.35 (6.46)	12.39 (11.06)	14.54 (12.54)				
BMI (Kg/m^2)	27.03 (3.97)	37.80 (0)	33.65 (6.42)	25.00 (1.97)	< 0.0001[a]	< 0.0001[a]	< 0.0001[a]	< 0.0001[a]
Fasting glucose (mmol/L)	11.10 (3.7)	5.9 (0)	8.42 (2.65)	7.66 (2.09)	< 0.0001[a]	< 0.0001[a]	0.69	0.001[a]
HbA1c (mmol/mol)	75.31 (16.74)	38 (0)	53.47 (13.24)	45.25 (6.43)	< 0.0001[a]	< 0.0001[a]	< 0.0001[a]	< 0.0001[a]
HbA1c (%)	9.0 (3.7)	5.6 (0)	7.0 (3.3)	6.3 (2.7)	< 0.0001[a]	< 0.0001[a]	< 0.0001[a]	< 0.0001[a]
Fasting C-Peptide (nmol/L)	0.32 (0.30)	2.27 (0)	0.70 (0.48)	0.36 (0.2)	< 0.0001[a]	< 0.0001[a]		
HOMA2B	24.22 (15.64)	219.3 (0)	58.21 (33.85)	41.64 (18.63)	< 0.0001[a]	< 0.0001[a]		
HOMA2IR	1.23 (0.96)	5.23 (0)	1.89 (1.27)	0.97 (0.47)	< 0.0001[a]	< 0.0001[a]		

	Male participants (243)							
**Cluster**	**SIDD**	**SIRD**	**MOD**	**MARD**	**p**	**p1**	**p: SIDD**	**p:MOD**
**Number (%)**	77 (31.69)	-	148 (60.91)	18 (7.4)				
Age at Diagnosis (y)	33.94 (7.86)	-	37.66 (6.76)	48.21 (3.76)	<0.0001[a]	<0.0001[a]	<0.0001[a]	0.09
Duration of diabetes (y)	20.77 (13.62)	-	11.74 (10.20)	13.64 (8.61)				
BMI (Kg/m^2)	26.85 (3.53)	-	34.85 (6.11)	32.30 (10.42)	<0.0001[a]	<0.0001[a]	<0.0001[a]	<0.0001[a]
Fasting glucose (mmol/L)	10.59 (3.55)	-	8.63 (2.60)	9.08 (3.14)	<0.0001[a]	<0.0001[a]	0.45	0.0002[a]
HbA1c (mmol/mol)	72.47 (16.55)	-	54.18 (13.61)	45.35 (6.09)	<0.0001[a]	<0.0001[a]	<0.0001[a]	0.0001[a]
HbA1c (%)	8.8 (3.7)		7.1 (3.4)	6.3 (2.7)	<0.0001[a]	<0.0001[a]	<0.0001[a]	0.0001[a]
Fasting C-Peptide (nmol/L)	0.32 (0.28)	-	0.78 (0.54)	0.65 (0.39)	<0.0001[a]	<0.0001[a]		

HOMA2B	25.52 (15.73)	-	58.59 (34.06)	49.50 (23.62)	<0.0001[a]	<0.0001[a]		
HOMA2IR	1.15 (0.86)	-	2.12 (1.50)	1.84 (1.02)	<0.0001[a]	<0.0001[a]		

	**Female participants (177)**							
**Cluster**	**SIDD**	**SIRD**	**MOD**	**MARD**	**p**	**p1**	**p: SIDD**	**p:MOD**
**Number (%)**	22 (12.43)	1 (0.56)	149 (84.18)	5 (2.82)				
Age at Diagnosis (y)	35.54 (7.10)	41.20(0)	36.33 (6.96)	42.98 (1.95)	0.088	0.084	0.40	0.24
Duration of diabetes (y)	19.49(12.15)	0.35 (-)	13.04 (11.84)	15.89 (21.23)				
BMI (Kg/m^2)	27.67 (5.30)	37.80 (0)	32.46 (6.50)	23.28 (0.87)	<0.0001[a]	<0.0001[a]	0.06	0.24
Fasting glucose (mmol/L)	12.90 (3.92)	5.90 (0)	8.20 (2.69)	6.96 (1.90)	<0.0001[a]	<0.0001[a]	0.10	0.35
HbA1c (mmol/mol)	85.23 (13.56)	38 (0)	52.75 (12.87)	44.87 (8.35)	<0.0001[a]	<0.0001[a]	0.09	0.0002[a]
HbA1c (%)	9.9 (3.4)	5.6 (0)	7.0 (3.3)	6.3 (2.9)	<0.0001[a]	<0.0001[a]	0.09	0.0002[a]
Fasting C-Peptide (nmol/L)	0.34 (0.32)	2.27 (0)	0.63 (0.39)	0.28 (0.21)	0.0015[a]	0.0016[a]		
HOMA2B	19.72 (14.81)	219.3 (0)	57.82 (33.76)	44.30 (17.39)	<0.0001[a]	<0.0001[a]		
HOMA2IR	1.52 (1.26)	5.23 (0)	1.67 (0.96)	0.82 (0.40)	<0.0001[a]	<0.0001[a]		

Note: Values are mean (SD), p-value by ANOVA, p1 adjusted for duration of diabetes. P: SIDD and P: MOD= p values for SIDD group and MOD group comparisons between WellGen and DIREVA respectively. Bonferroni corrected significant p-values are indicated with a.

ESM table 10: Characteristics of participants enrolled in the DIREVA study by clusters for all participants, men and women.

	Ahmedabad				
	All (187)				
**Cluster**	**SIDD**	**SIRD**	**MOD**	**MARD**	**p**
**Number (%)**	106 (56.68)	0 (0)	62 (33.15)	19 (10.16)	
Age at Diagnosis (y)	37.05(6.66)		34.84 (6.79)	42.05(3.34)	0.0002[a]
BMI (Kg/m^2)	26.76(3.34)		29.99 (3.79)	25.06(2.68)	<0.0001[a]
Fasting glucose (mmol/L)	13.03 (4.53)		8.48 (3.26)	6.66 (2.13)	<0.0001[a]
HbA1c (mmol)	88.25(22.94)		53.6 (11.18)	48.29(8.91)	<0.0001[a]
HbA1c (%)	10.2 (4.3)		7.1 (3.2)	6.5 (2.9)	<0.0001[a]
Fasting C-Peptide (nmol/L)	0.43 (0.20)		0.48 (0.18)	0.44 (0.20)	0.282
HOMA2B	22.83(18.63)		48.48 (30.84)	64.38(39.32)	<0.0001[a]
HOMA2IR	1.66(1.22)		1.34(0.86)	1.06(0.52)	0.032
	**Ahmedabad: Male participants (138)**				
**Cluster**	**SIDD**	**SIRD**	**MOD**	**MARD**	**p**
**Number**	85 (61.59)	0 (0)	36 (26.08)	17 (12.32)	
Age at Diagnosis (y)	36.63(6.85)		34.11(6.44)	41.71(3.37)	<0.0001[a]
BMI (Kg/m^2)	26.5(3.32)		30.66 (2.97)	25.33(2.68)	<0.0001[a]
Fasting glucose (mmol/L)	12.66 (4.45)		8.05 (3.24)	6.50 (1.71)	<0.0001[a]
HbA1c (mmol)	86.38(23.16)		52.09 (12.55)	47.22(8.79)	<0.0001[a]
HbA1c (%)	10.0 (4.3)		6.9 (3.3)	6.5 (3.0)	<0.0001[a]
Fasting C-Peptide (nmol/L)	0.43 (0.22)		0.51 (0.18)	0.45 (0.21)	0.218
HOMA2B	24.17(19.34)		56.15 (35.34)	63.91(36.8)	<0.0001[a]
HOMA2IR	1.62(1.23)		1.4 (0.91)	1.1(0.54)	0.168

	Ahmedabad: Female participants (49)				
Cluster	SIDD	SIRD	MOD	MARD	p
**Number (%)**	21 (42.86)	0 (0)	26 (53.06)	2 (4.10)	
Age at Diagnosis (y)	38.76(5.66)		35.85 (7.24)	45(0)	0.084
BMI (Kg/m^2)	27.83(3.31)		29.07 (4.59)	22.76(1.68)	0.093
Fasting glucose (mmol/L)	14.53 (4.66)		8.89 (3.34)	7.99 (5.57)	<0.0001[a]
HbA1c (mmol)	95.79(20.86)		55.63 (9.05)	57.38(3.09)	<0.0001[a]
HbA1c (%)	10.9 (4.1)		7.3 (3.0)	7.5 (2.4)	<0.0001[a]
Fasting C-Peptide (nmol/L)	0.40 (0.14)		0.44 (0.18)	0.31 (0.05)	0.480
HOMA2B	17.41(14.6)		37.87 (19.23)	68.35(78.28)	<0.0001[a]
HOMA2IR	1.82(1.19)		1.25 (0.8)	0.75(0.06)	0.094

Note: Values are mean (SD), p-value by ANOVA. Bonferroni corrected significant p-values are indicated in bold.

ESM table 11. Characteristics of participants enrolled in the Ahmedabad study by clusters for all participants, for men and women

	Assam					
	All (205)					
**Cluster**	**SIDD**	**SIRD**	**MOD**	**MARD**	**p**	**p1**
**Number (%)**	138 (66.66)	3 (1.40)	48 (23.20)	16 (7.72)		
Age at Diagnosis (y)	32.14 (5.3)	34 (6.56)	31.48 (5.44)	35.38 (3.1)	0.069	0.112
Duration of diabetes (y)	3.66 (3.83)	4.26 (5.04)	3.01 (3.15)	1.81 (2.00)	0.209	-
BMI (Kg/m^2)	22.68 (3.43)	17.6 (3.5)	26.25 (3.44)	21.79 (2.95)	<0.0001[a]	<0.0001[a]
Fasting glucose (mmol/L)	11.89 (4.14)	29.11 (3.32)	6.84 (2.08)	6.18 (1.27)	<0.0001[a]	<0.0001[a]
HbA1c (mmol)	97.06 (26.51)	105.46 (52.95)	52.44 (13.91)	39.62 (7.9)	<0.0001[a]	<0.0001[a]
HbA1c (%)	11.0 (4.6)	11.8 (7.0)	6.9 (3.4)	5.8 (2.8)	<0.0001[a]	<0.0001[a]
Fasting C-Peptide (nmol/L)	0.38 (0.32)	0.07 (0.01)	0.57 (0.39)	0.33 (0.25)	0.002[a]	0.002[a]
HOMA2B	24.26 (18.7)	3.2 (0)	71.03 (39.28)	58.6 (31.49)	<0.0001[a]	<0.0001[a]
HOMA2IR	1.4 (1.19)	30.3 (0)	1.45 (0.92)	0.88 (0.52)	<0.0001[a]	<0.0001[a]
	Assam: Male participants (135)					
**Cluster**	**SIDD**	**SIRD**	**MOD**	**MARD**	**p**	**p1**
**Number (%)**	99 (73.33)	1 (0.74)	21 (15.55)	14 (10.37)		
Age at Diagnosis (y)	32.53 (4.93)	40 (NA)	32.86 (4.96)	35.36 (3.32)	0.096	0.225
Duration of diabetes (y)	3.20 (3.57)	0.15 (NA)	2.23 (3.06)	1.72 (1.96)	0.267	-
BMI (Kg/m^2)	22.73(3.53)	15.35 (NA)	28.03(2.44)	22.39(2.52)	<0.0001[a]	<0.0001[a]
Fasting glucose (mmol/L)	11.59 (4.35)	31.97 (NA)	5.98 (1.30)	6.29 (1.29)	<0.0001[a]	<0.0001[a]
HbA1c (mmol)	94.72(27.32)	163.39 (NA)	48.22(13.56)	39.81(8.46)	<0.0001[a]	<0.0001[a]
HbA1c (%)	10.8 (4.6)	17.1 (NA)	6.5 (3.4)	5.8 (2.9)	<0.0001[a]	<0.0001[a]
Fasting C-Peptide (nmol/L)	0.36 (0.28)	0.08 (NA)	0.64 (0.40)	0.35 (0.26)	0.001[a]	0.001[a]
HOMA2B	25.32(19.57)	3.2 (NA)	91.66(39.73)	58.89(33.75)	<0.0001[a]	<0.0001[a]

HOMA2IR	1.35(1.14)	30.3 (NA)	1.56(0.9)	0.92(0.54)	<0.0001[a]	<0.0001[a]
	**Assam: Female participants (70)**					
**Cluster**	**SIDD**	**SIRD**	**MOD**	**MARD**	**p**	**p1**
**Number (%)**	39 (54.16)	2 (2.77)	27 (37.5)	2 (2.77)		
Age at Diagnosis (y)	31.15 (6.1)	31 (5.66)	30.41 (5.65)	35.5 (0.71)	0.689	0.562
Duration of diabetes (y)	4.82 (4.27)	6.32 (5.04)	3.61 (3.14)	2.44 (3.02)	0.467	-
BMI (Kg/m^2)	22.58 (3.19)	18.73 (4.11)	24.86 (3.49)	17.61 (2.79)	0.001[a]	0.002[a]
Fasting glucose (mmol/L)	12.68 (3.50)	27.69 (3.13)	7.50 (2.34)	5.41 (1.06)	<0.0001[a]	<0.0001[a]
HbA1c (mmol)	103 (23.61)	76.5 (23.96)	55.72 (13.52)	38.25 (0.77)	<0.0001[a]	<0.0001[a]
HbA1c (%)	11.6 (4.3)	9.2 (4.3)	7.3 (3.4)	5.6 (2.2)	<0.0001[a]	<0.0001[a]
Fasting C-Peptide (nmol/L)	0.44 (0.40)	0.07 (0)	0.51 (0.38)	0.21 (0.18)	0.333	0.381
HOMA2B	21.56 (16.2)	3.2 (0)	54.99 (31.04)	56.55 (7.28)	<0.0001[a]	<0.0001[a]
HOMA2IR	1.53 (1.32)	30.3 (0)	1.37 (0.95)	0.62 (0.26)	<0.0001[a]	<0.0001[a]

Note: Values are mean (SD), p-value by ANOVA, p1 adjusted for duration of diabetes. Bonferroni corrected significant p-values are indicated with a.

ESM Table 12. Characteristics of participants enrolled in the Assam study by clusters for all participants, men and women.

ESM Figures

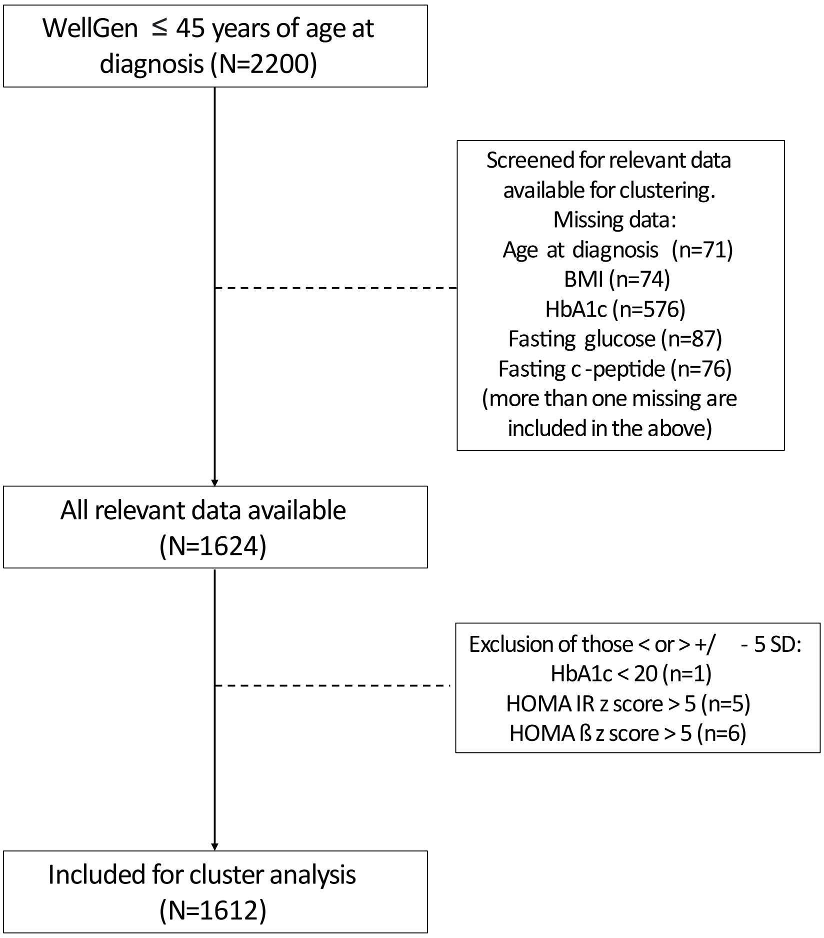

ESM figure 1: Flowchart for the WellGen cohort.

2a

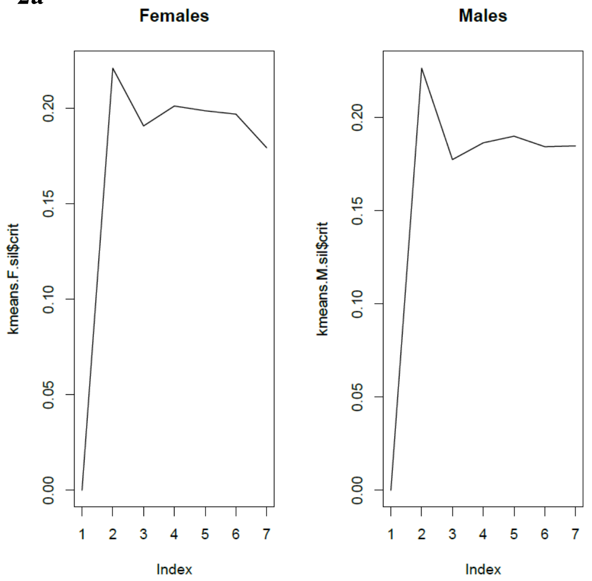

2b

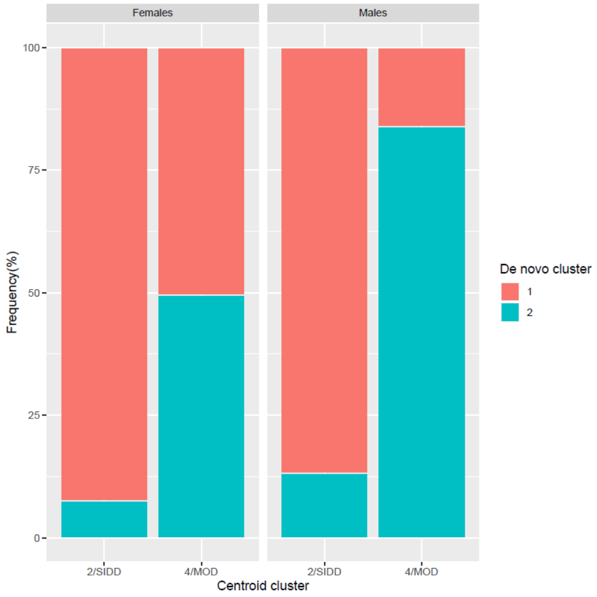

ESM figure 2. 2a. Silhouette plot showing optimal number of clusters in the WellGen cohort. 2b. Nearest centroid vs k-means clustering: Overlap of distribution of patients in the two largest clusters derived using two methods. De novo k-means clustering showed two clusters, with patients in cluster 1 showing as 88.8 % overlap with those in SIDD (ALL: 88.88%, Men: 86.8%, Women: 92.4%) whereas patients in cluster 2 patients showed a 62.5% overlap with MOD (ALL: 62.5%, Men: 83.9% and Women: 49.5%).

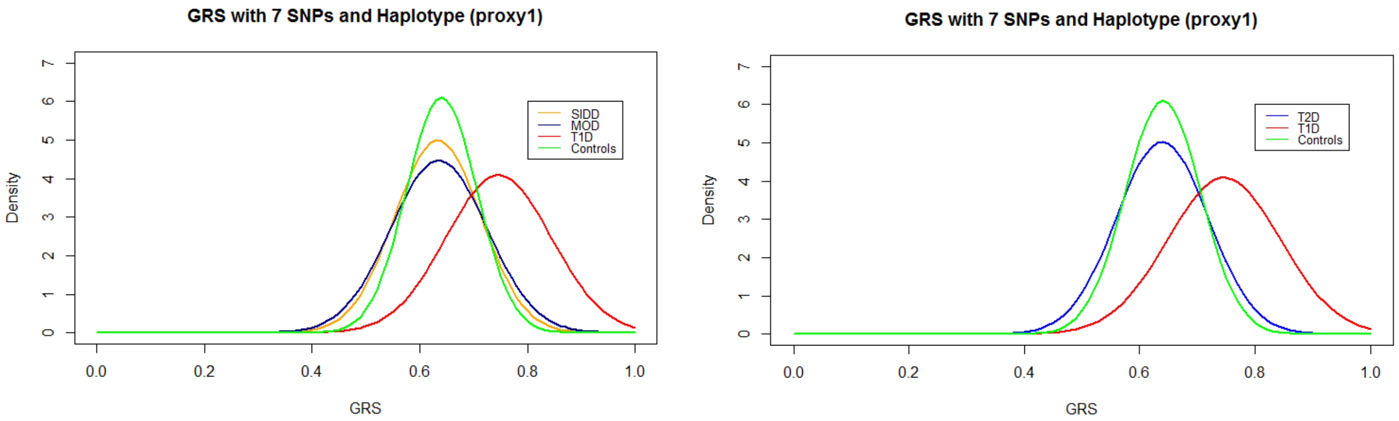

ESM figure 3. Density plot of genetic risk score distributions in T1D, SIDD, MOD and controls in the WellGen study.

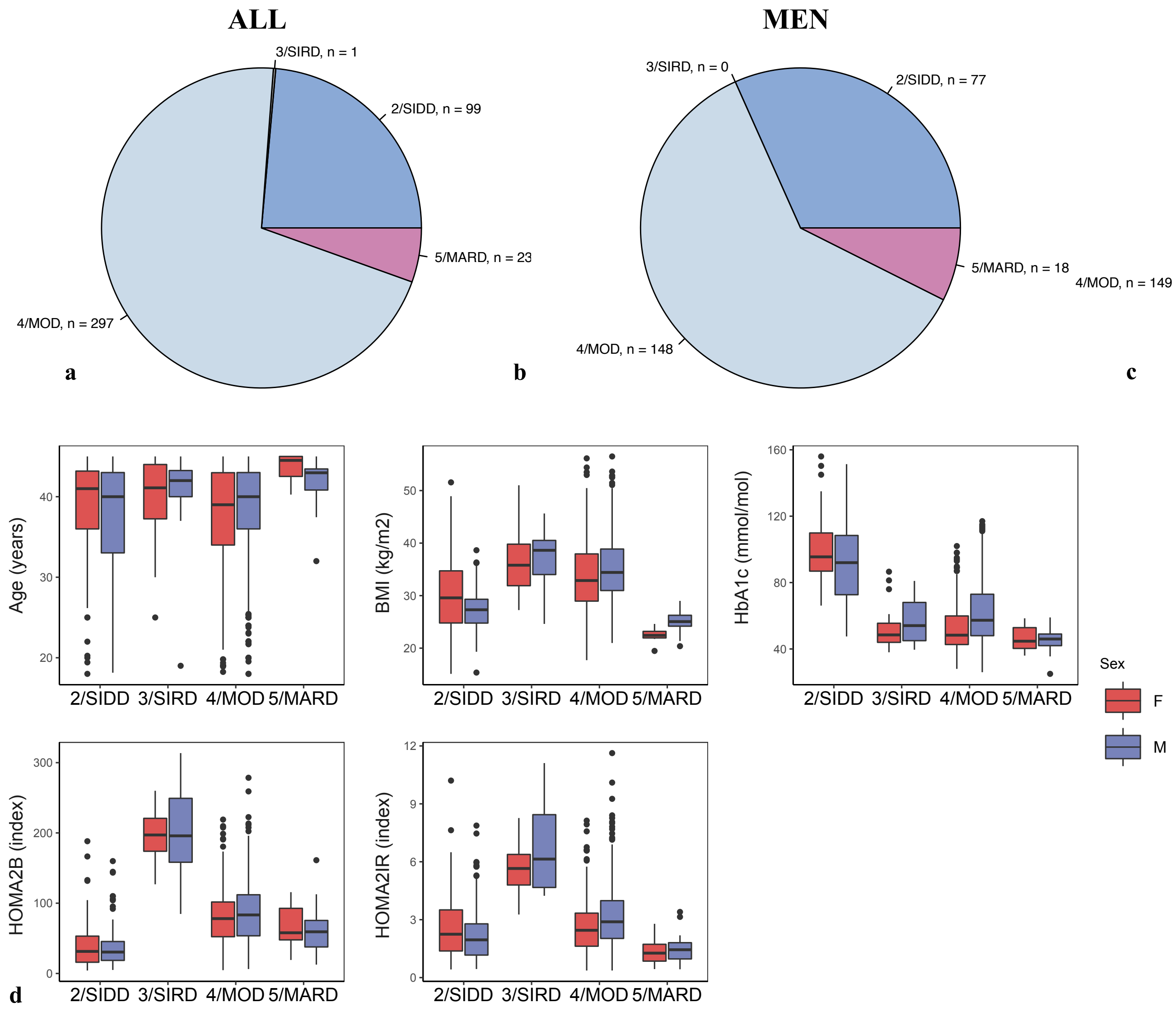

ESM figure 4. Patient distribution in various predefined clusters. (a) Distribution of DIREVA patients (n = 424) (b) distribution of men with diabetes from the DIREVA study (n = 243) (c) distribution of women with diabetes from the DIREVA study (n =177). (d) Box plots of cluster characteristics of patients in the DIREVA study. Distribution of age at diagnosis, BMI, HbA1c, HOMA2-B and HOMA2-IR in the DIREVA study for each cluster. The central line within the box represents the median and the upper and lower limits of the box represent the interquartile range. The whiskers are the most extreme values within 1.5× the interquartile range from the first and second quartiles. k-means clustering was done separately for men and women; data is shown for each sex separately. SIDD = severe insulin-deficient diabetes, SIRD = resistant diabetes, MOD = mild obesity-related-related diabetes, MARD = mild age-related diabetes. HOMA2-B = homeostatic model assessment 2 estimates of beta cell function. HOMA2-B = homeostatic model assessment 2 estimates insulin resistance.

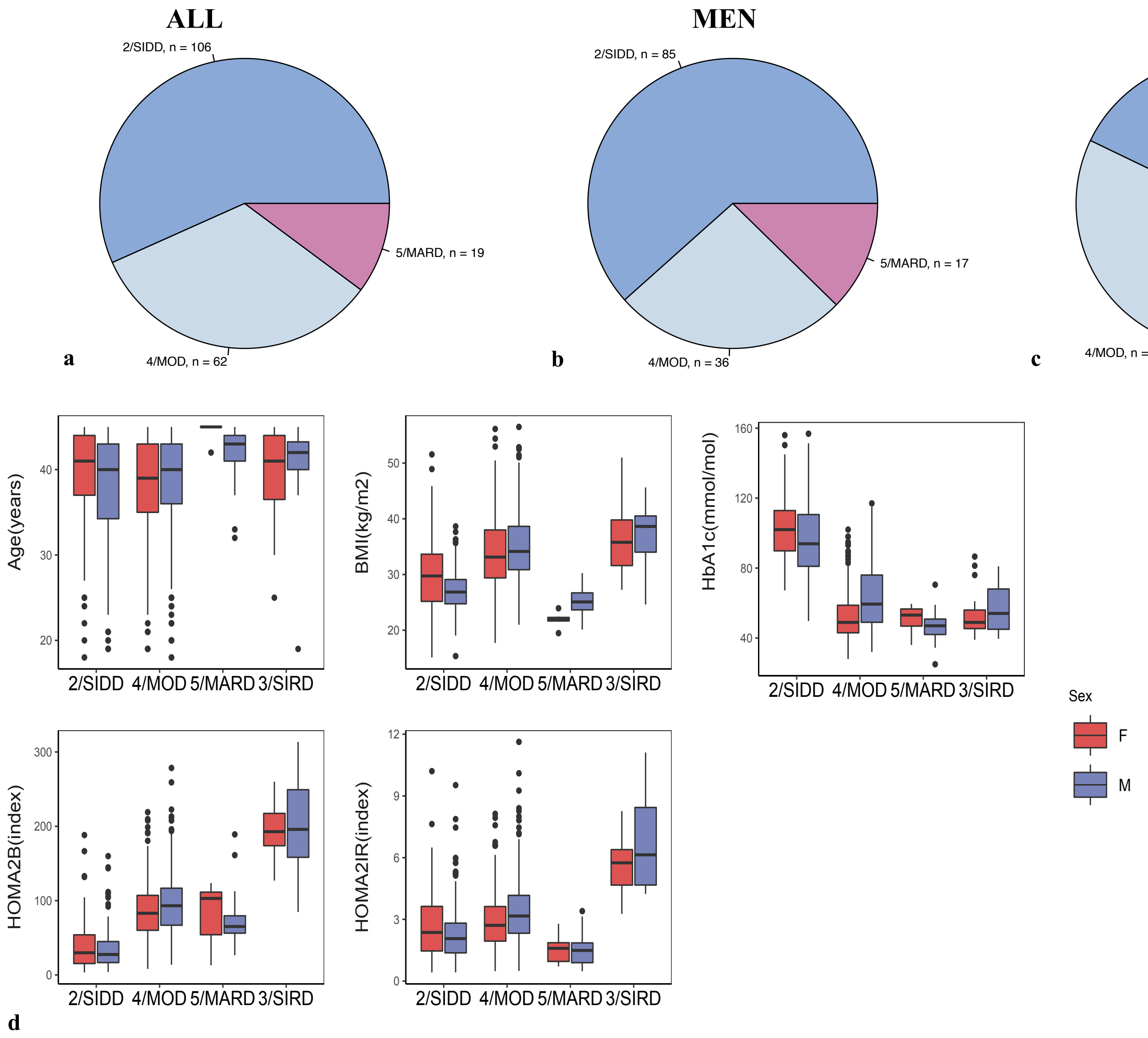

ESM figure 5. Distribution of participants from the Ahmedabad study in the predefined clusters. (a) Distribution of patients (n =) (b) distribution of women with diabetes (n =) (c) distribution of men with diabetes (n =) (d) Box plots of cluster characteristics in the Ahmedabad cohort. Distribution of age at diagnosis, BMI, HbA1c, HOMA2-B and HOMA2-IR in the Ahmedabad study for each cluster. The central line within the box represents the median and the upper and lower limits of the box represent the interquartile range. The whiskers are the most extreme values within 1.5× the interquartile range from the first and second quartiles. k-means clustering was done separately for men and women; data is shown for each sex separately. SIDD = severe insulin-deficient diabetes, SIRD = resistant diabetes, MOD = mild obesity-related-related diabetes, MARD = mild age-related diabetes. HOMA2-B = homeostatic model assessment 2 estimates of beta cell function. HOMA2-B = homeostatic model assessment 2 estimates insulin resistance.

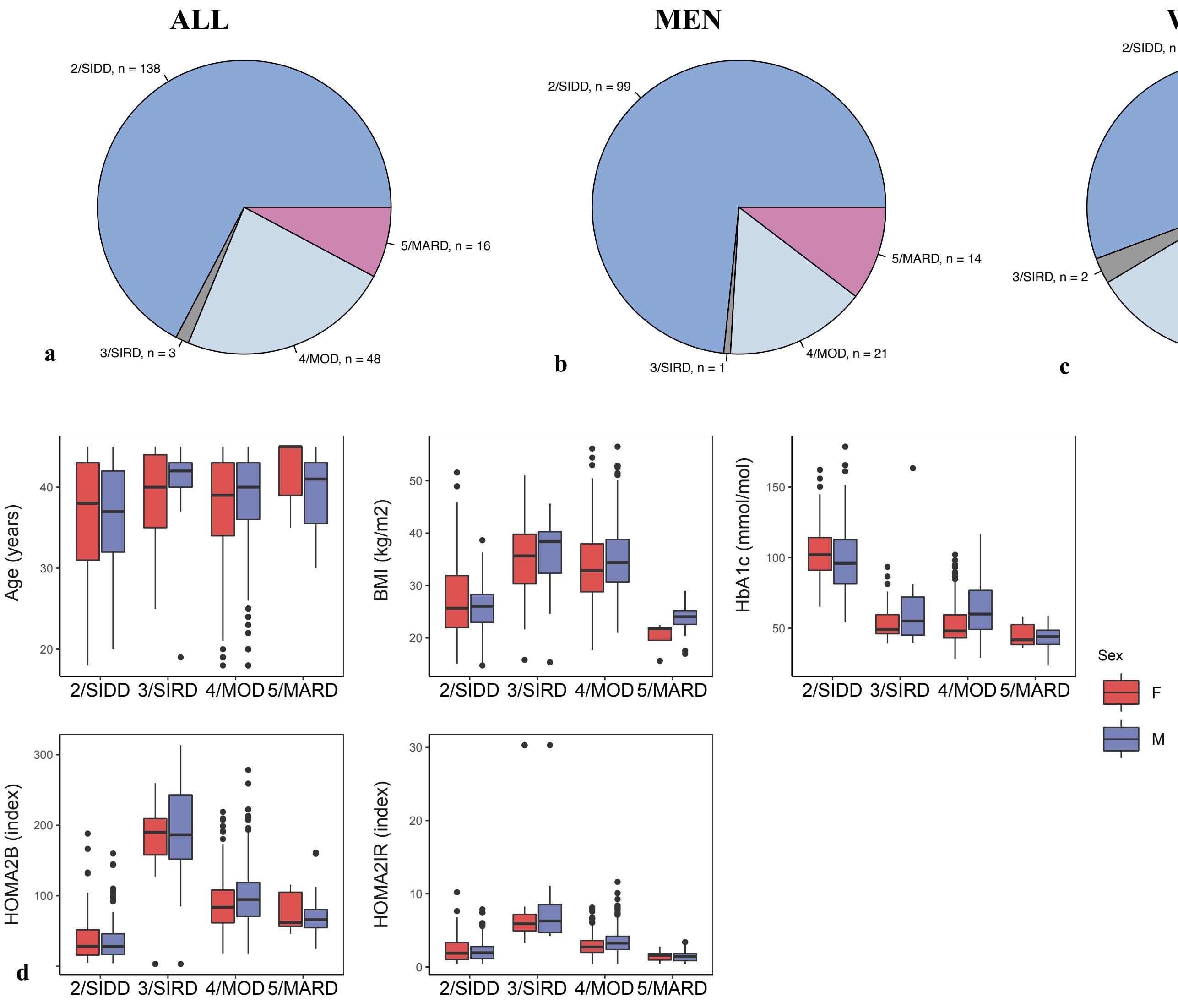

ESM Figure 6. Distribution of participants from the Assam study in the predefined clusters. (a) Distribution of patients (n =) (b) distribution of women with diabetes (n =) (c) distribution of men with diabetes (n =) (d) Box plots of cluster characteristics in the Assam cohort. Distribution of age at diagnosis, BMI, HbA1c, HOMA2-B and HOMA2-IR in the Assam study for each cluster. The central line within the box represents the median and the upper and lower limits of the box represent the interquartile range. The whiskers are the most extreme values within 1.5× the interquartile range from the first and second quartiles k-means clustering was done separately for men and women; data is shown for each sex separately. SIDD = severe insulin-deficient diabetes, SIRD = resistant diabetes, MOD = mild obesity-related-related diabetes, MARD = mild age-related diabetes. HOMA2-B = homeostatic model assessment 2 estimates of beta cell function. HOMA2-B = homeostastic model assessment 2 estimates insulin resistance.
